# Loss of *Bacteroides thetaiotaomicron* bile acid-altering enzymes impacts bacterial fitness and the global metabolic transcriptome

**DOI:** 10.1128/spectrum.03576-23

**Published:** 2023-11-29

**Authors:** Arthur S. McMillan, Matthew H. Foley, Caroline E. Perkins, Casey M. Theriot

**Affiliations:** 1 Department of Biological Sciences, Genetics Program, College of Science, North Carolina State University, Raleigh, North Carolina, USA; 2 Department of Population Health and Pathobiology, College of Veterinary Medicine, North Carolina State University, Raleigh, North Carolina, USA; 3 Department of Food, Bioprocessing and Nutrition Sciences, North Carolina State University, Raleigh, North Carolina, USA; Wayne State University, Detroit, Michigan, USA

**Keywords:** bile acids, *Bacteroides thetaiotaomicron*, bile salt hydrolase, hydroxysteroid dehydrogenase, carbohydrates, amino acids, nutrient limitation

## Abstract

*Bacteroides thetaiotaomicron* (*B. theta*) is a Gram-negative gut bacterium that encodes enzymes that alter the bile acid pool in the gut. Primary bile acids are synthesized by the host liver and are modified by gut bacteria. *B. theta* encodes two bile salt hydrolases, as well as a hydroxysteroid dehydrogenase. We hypothesize that *B. theta* modifies the bile acid pool in the gut to provide a fitness advantage for itself. To investigate each gene’s role, different combinations of genes encoding bile acid-altering enzymes (*bshA*, *bshB*, and *hsdhA*) were knocked out by allelic exchange, including a triple KO. Bacterial growth and membrane integrity assays were done in the presence and absence of bile acids. To explore if *B. theta’s* response to nutrient limitation changes due to the presence of bile acid-altering enzymes, RNA sequencing (RNASeq) analysis of wild type (WT) and triple knockout (KO) strains in the presence and absence of bile acids was done. WT *B. theta* is more sensitive to deconjugated bile acids such as cholate, chenodeoxycholate (CDCA), and deoxycholate (DCA) compared with the triple KO. These deconjugated bile acids also decreased membrane integrity of both WT and triple KO. The presence of *bshB* is detrimental to growth in conjugated forms of CDCA and DCA. RNASeq analysis also showed that bile acid exposure impacts multiple metabolic pathways in *B. theta*, but DCA significantly increases expression of many genes in carbohydrate metabolism, specifically those in polysaccharide utilization loci or PULs, in nutrient-limited conditions. This study suggests that bile acids *B. theta* encounters in the gut may signal the bacterium to increase or decrease its utilization of carbohydrates. Further study looking at the interactions between bacteria, bile acids, and the host may inform rationally designed probiotics and diets to ameliorate inflammation and disease.

**IMPORTANCE:**

Recent work on bile salt hydrolases (BSHs) in Gram-negative bacteria, such as Bacteroides, has primarily focused on how they can impact host physiology. However, the benefits bile acid metabolism confers to the bacterium that performs it are not well understood. In this study, we set out to define if and how *Bacteroides thetaiotaomicron* (*B. theta*) uses its BSHs and hydroxysteroid dehydrogenase to modify bile acids to provide a fitness advantage for itself *in vitro* and *in vivo*. Genes encoding bile acid-altering enzymes were able to impact how *B. theta* responds to nutrient limitation in the presence of bile acids, specifically carbohydrate metabolism, affecting many polysaccharide utilization loci. This suggests that *B. theta* may be able to shift its metabolism, specifically its ability to target different complex glycans including host mucin, when it comes into contact with specific bile acids in the gut.

## INTRODUCTION

The microbiota of the intestinal tract is made up of a diversity of microbes that interact with the host and each other. Bile acid metabolism represents an important function that mediates the relationship between the host and the gut microbiota (1 – 4). Bile acids are synthesized from cholesterol in the liver, stored in the gallbladder, and secreted when the host eats a meal (5). Their role during digestion is to act as surfactants allowing for the absorption of dietary fats and vitamins. Once they reach the terminal ileum, ~95% of bile acids is reabsorbed through enterohepatic circulation (6, 7). Bile acids directly interact with the host through receptors like Farnesoid X Receptor and Pregnane X Receptor that regulate the production of bile acids made by the host (1, 8) but can also influence other aspects of host physiology, such as immunity by modulating T_reg_ and T_H_17 cell differentiation (8, 9).

Bile acids also shape the gut microbiome’s composition due to their antimicrobial and detergent-like properties (4). Distinct changes to the bile acid pool and microbiome are associated with a wide variety of diseases including *Clostridioides difficile* infection (CDI) (10), inflammatory bowel disease (11), metabolic syndrome (12), and cancer (12, 13). However, the mechanisms that dictate the bacterial-bile acid relationship are not fully understood. There is great interest in defining these mechanisms in order to leverage the gut microbiota’s ability to alter the bile acid pool, promoting host health.

During their intestinal transit, bile acids are modified by gut bacteria which changes the chemistry and diversity of the bile acid pool. The gut microbiota encodes a variety of bile acid-altering enzymes that act on either the sterol core or the amino acid conjugated to it. Bile salt hydrolases (BSHs) are traditionally known to cleave the glycine or taurine amino acid from conjugated bile acids, yet recent work has prescribed a new function for BSHs: the production of bile acids conjugated with a variety of additional amino acids, collectively referred to as microbial conjugated bile acids (MCBAs) (14 – 16). Historically, it was proposed that the primary role of BSHs in bacteria was to detoxify conjugated bile acids thereby promoting bacterial colonization in the harsh gut environment (17). It has also been hypothesized that BSHs are able to provide nutrients in the form of the liberated amino acid (either taurine or glycine) for the microbe that encodes them or the surrounding gut microbiota (18). However, a recent study using Gram-positive lactobacilli found that some bile acids became more toxic to the bacterium after deconjugation (19). The bile acid chemical structure and the different BSH substrate preferences together were able to influence *in vitro* and *in vivo* growth of the different lactobacilli strains (19). Hydroxysteroid dehydrogenases (HSDHs) were also thought to detoxify bile acids as well as use bile acids as an electron donor for the electron transport chain (20 – 22), but this enzyme has been studied even less.

Recent work on BSHs in Gram-negative bacteria, such as *Bacteroides*, has primarily focused on how they can impact host physiology (23, 24). Bile acid metabolism has been implicated in fecal microbiota transplants for treatment of recurrent CDI, cholestatic liver injury, and diabetes in bariatric surgery recipients, as well as in a member of the ability of *Bacteroidales* to ameliorate hepatic fibrosis (25 – 28). However, the benefits bile acid metabolism confers to the bacterium that performs it are not well understood. *Bacteroides thetaiotaomicron* (*B. theta*) is a Gram-negative organism, which encodes multiple genes encoding bile acid-altering enzymes (29). This organism is widely studied due to its unique carbohydrate utilization ability and for this reason has many genetic tools (30, 31). It is also a prominent member of the human intestinal tract (32). *B. theta* encodes two putative BSHs (BT_1259 referred to in this paper as BSHa and BT_2086 referred to in this paper as BSHb) (29). The majority of bile acid deconjugation in *B. theta* is done by BSHb (24), although there is evidence that BSHa may act on high concentrations of TCA and GCA (33). The BSHs encoded by *B. theta* have a limited capacity to produce MCBAs thus far, but this requires further study (34). *B. theta* also encodes a 7α-hydroxysteroid dehydrogenase or HSDH (BT_1911 referred to in this paper as HSDHa) that has not been well studied. αHSDHs act on the sterol core of bile acids to convert the hydroxyl to a ketone that can, in combination with other enzymes, result in the epimerization of hydroxyl groups or remove them entirely (35). The HSDH encoded by *B. theta* has activity on two major bile acids which contain a 7-hydroxyl group by oxidizing the 7-hydroxyl to a ketone (ex. converting CDCA to 7-oxo LCA) (36).

In this study, we set out to define if and how *B. theta* uses its BSHs and HSDH to modify bile acids to provide a fitness advantage for itself *in vitro* and *in vivo*. To address this, we created gene knockouts, single and multiple combinations of both BSHs (*bshA* and *bshB*) and HSDH (*hsdhA*) in *B. theta*. We found that *B. theta* could grow in higher concentrations of taurine- and glycine-conjugated CA compared with CDCA and DCA. It was also more sensitive to deconjugated bile acids in growth and in membrane integrity assays. *bshB* was detrimental to *B. theta* growth in the presence of conjugated bile acids that it could act on. RNASeq analysis was done on WT and triple KO strains in rich and minimal media (MM) in the presence and absence of bile acids to decipher the relationship between these enzymes and how they affect the global metabolic transcriptome. Genes encoding bile acid-altering enzymes were able to impact how *B. theta* responds to nutrient limitation in the presence of bile acids, specifically carbohydrate metabolism, affecting many polysaccharide utilization loci (PULs). This suggests that *B. theta* may be able to shift its metabolism, specifically its ability to target different complex glycans including host mucin, when it comes into contact with specific bile acids in the gut. Bacterial fitness of the WT and triple KO strains showed modest differences *in vivo*, in different mouse models that varied in their bile acid pool. This work will aid in our understanding of how to rationally manipulate the bile acid pool and the microbiota to exploit carbohydrate metabolism in the context of inflammation and other GI diseases.

## MATERIALS AND METHODS

### Bacterial strains and culture conditions

The background for mutant strains is *B. thetaiotaomicron* VPI-5482 ∆*tdk* (WT) that was acquired from the Martens lab at the University of Michigan. *B. theta* strains were grown statically at 37°C in a Coy anaerobic chamber (2.5% H_2_/10% CO_2_/88.5% N2) in TYG medium (10 g/L tryptone, 5 g/L yeast extract, 4 g/L D-glucose, 100 mM KH_2_PO4, 8.5 mM [NH_2_]_4_SO_4_, 15 mM NaCl, 5.8 µM vitamin K3, 1.44 µM FeSO4⋅7H_2_O, 1 mM MgCl_2_, 1.9 µM hematin, 0.2 mM L-histidine, 3.69 nM vitamin B12, 208 µM L-cysteine, and 7.2 µM CaCl_2_⋅2H_2_O) or in MM (0.5% D-glucose, 100 mM KH_2_PO4, 8.5 mM [NH_2_]_4_SO_4_, 15 mM NaCl, 5.8 µM vitamin K3, 1.44 µM FeSO4⋅7H_2_O, 1 mM MgCl_2_, 1.9 µM hematin, 0.2 mM L-histidine, 3.69 nM vitamin B12, 208 µM L-cysteine, and 7.2 µM CaCl_2_⋅2H_2_O). Solid media for *B. theta* were Brain heart infusion (Difco) agar supplemented with 10% Horse Blood (LAMPIRE) (BHI-HB). Liquid *E. coli* cultures were grown at 37°C aerobically with shaking in LB (Sigma). Solid media for *E. coli* cultures were grown statically at 37°C aerobically on LB agar. Selective drug concentrations were consistent across solid and liquid media and supplemented during appropriate selection steps (150 µg/mL ampicillin, 200 µg/mL gentamicin, 25 µg/mL erythromycin, and 200 µg/mL FUDR).

### Deletion of *bsh* and *hsdh* genes from *B. thetaiotaomicron*

Clean in-frame deletions of bile-altering enzymes were generated using a counterselectable allelic exchange procedure (31). Briefly, 750-basepair regions flanking each gene were amplified by PCR and Hi-Fi DNA Assembly (NEB) was used to clone into pExchange-*tdk*. Plasmids were verified using Sanger sequencing and electroporated into *E. coli* S17 and selected for on solid media supplemented with ampicillin to function as a conjugal donor. An approximately equivalent donor-to-recipient ratio was used for conjugation. Merodiploids were selected after conjugation on solid *B. theta* media supplemented with gentamicin and erythromycin. A pool of merodiploids was grown in liquid media in the absence of antibiotic to allow for excision of the plasmid from the genome. Successful transformants were selected for solid *B. theta* media containing FUDR to select against colonies that still contain the plasmid. Single colonies were then verified as successful knockouts using PCR and Sanger sequencing.

### Minimum inhibitory concentrations

Bile acid, or BA, tolerance measured by MIC was adapted from a previously established bile tolerance assay (19, 37). Overnight cultures (~10^10^ CFUs/mL) were inoculated at approximately 10^8^ CFUs/mL into tryptone yeast glucose (TYG) containing a range of BA concentrations. Cultures were anaerobically incubated for a total of 24 h at 37°C. At both 12 h and 24 h, cultures were serially diluted in phosphate buffered saline (PBS) and plated on brain heart infusion plus 10% horse blood (BHI-HB) to determine if the concentration of BA tested inhibited growth to prevent at least one doubling of colonies after either 12 or 24 h of growth.

### Propidium iodide staining

Overnight cultures (~10^10^ CFUs/mL) were diluted to an optical density or OD_600_ of 0.1 and incubated until an OD_600_ of 0.8 was reached. The following concentrations: 4 mM TCA, 4 mM GCA, 5 mM CA, 1.25 mM TCDCA, 1.25 mM GCDCA, 0.3125 mM CDCA, 0.6375 mM TDCA, 0.6375 mM GDCA, 0.3125 mM DCA, or 150 µM SDS, were then introduced and incubated at 37°C for 30 min. Following this incubation, bacteria were stained with 20 µM PI using previously described methods (10, 11). Fluorescence of PI was measured using a TECAN INFINITE F200 (excitation = 560 nm, emission = 600 nm) and was normalized to the OD_600_ of the culture prior to introduction of bile acids.

### *B. theta* tolerance to subinhibitory concentrations of bile acids

Overnight cultures (~10^10^ CFUs/mL) were inoculated 1% into TYG containing a high yet survivable concentration of bile acid that results in approximately 10^10^ CFUs/mL after 12 h (4 mM TCA, 4 mM GCA, 5 mM CA, 1.25 mM TCDCA, 1.25 mM GCDCA, 0.3125 mM CDCA, 0.6375 mM TDCA, 0.6375 mM GDCA, and 0.3125 mM DCA). Cultures were anaerobically incubated for 24 h at 37°C. At both 12 h and 24 h, cultures were serially diluted in PBS and plated on BHI-HB to determine the effect each bile acid had on the fitness of *B. theta* with and without bile-altering enzyme(s).

### RNA extraction from liquid *B. theta* cultures

RNA extraction protocol was adapted from a previously optimized workflow (38). Overnight cultures were inoculated 1% into TYG or MM supplemented with 4 mM TCA, 4 mM GCA, 2.5 mM CA, 1.25 mM TCDCA, 1.25 mM GCDCA, 0.3125 mM CDCA, 0.637 5 mM TDCA, 0.6375 mM GDCA, and 0.156 mM DCA and allowed to reach mid-log (~1.6 OD) and were fixed 1:1 in a 50/50 ethanol/acetone solution and frozen at −80°C. Fixed cultures were thawed on ice, and the pellet was resuspended in 1:100 beta-mercaptoethanol. Pellets were then resuspended in TRIzol Reagent (Thermo) and incubated for 20 min. Chloroform was then added and vigorously inverted before incubation at room temperature for 20 min. The samples were centrifuged at 14,000 rpm at 4°C for 20  min. The aqueous phase (~650 µL) was then added to 650 µL of isopropanol that had been supplemented with 5 µg/mL glycogen. Samples were vortexed and incubated on ice for 20 min and then were centrifuged at 4°C for 30 min. Pellets were washed three times with 70% ethanol and then dissolved in sterile deionized water. The RNA was treated with Turbo DNase (Thermo Fisher, AM2239); the protocol was modified by increasing the amount of enzyme to 5 µL per sample. After 30 min of incubation in a heat block at 37°C, 2 µL of Turbo DNase enzyme was added to each sample for a further 30 min of incubation. The RNA was then column purified according to the manufacturer’s instructions (Zymo). Final concentrations of RNA were determined via Qubit RNA high-sensitivity assay kit.

### Quantitative reverse transcription PCR

RNA from liquid cultures of *B. theta* WT was used as template in reverse transcription reactions using the Murine Moloney Leukemia Virus Reverse Transcriptase (Thermo) following the manufacturer’s protocol. The resulting cDNA was diluted in deionized water such that approximately 10 ng would be used as template for quantitative PCR with the SsoAdvanced Universal SYBR Green Supermix (Bio-Rad). Each gene/bile acid combination assayed was analyzed using the ∆∆Ct method by comparison to the housekeeping gene 16S rRNA and a control culture grown without bile acid (39).

### RNASeq analysis

rRNA depletion and RNA sequencing using SP lanes on an Illumina NovaSeq 6000 were performed by the Roy J. Carver Biotechnology Center at the University of Illinois at Urbana-Champaign. Reads were mapped to *B. thetaiotaomicron* VPI-5482 (ASM1106v1) using Salmon with default settings. Differential expression analysis was performed in R using the DESeq2 package. Genes with less than 10 total reads were filtered. For comparisons between WT and triple KO strains of *B. theta*, the effect of the nutrient limitation as well as the change in the effect of nutrient limitation due to the genotype was analyzed via the DESeq2 package. For comparisons in growth of bile acids, the effect of the addition of bile acid as well as the change in effect of bile acid stress due to nutrient limitation was analyzed via the DESeq2 package. Kegg annotations were downloaded directly from KEGG database, and further annotation utilized BlastKOALA of translated nucleotide sequences to determine K numbers for annotation of unannotated genes. Target substrates and gene annotation of PULs were annotated using CAZy’s PULDB (http://www.cazy.org/PULDB/) and from multiple publications for deeper annotation (40 – 47).

### Protein cloning and expression

Codon-optimized BT_1911 and BT_1259 were synthesized from IDT and amplified by PCR with the Phusion Flash High-Fidelity PCR Master Mix (Thermo) using the custom oligonucleotide primers (IDT) listed in the primer table. Amplicons were purified using the QIAquick PCR Purification kit (Qiagen) and were cloned into the pETite C-His vector (Lucigen) with a C-terminal hexahistidine tag. The resulting plasmids were purified using the Monarch Plasmid Miniprep Kit (NEB) and sequenced. The expression plasmids were transformed into *E. coli* Rosetta (DE3) pLysS and were cultured at 37°C shaking overnight in LB broth supplemented with 30 µg mL^−1^ kanamycin (Kan) and 20 µg mL^−1^ chloramphenicol (Cam) and then in Terrific Broth (TB) supplemented with the same antibiotics for protein overexpression. Overexpression cultures were grown at 37°C shaking in 1 L of Terrific Broth with Kan and Cam until an OD_600_ ~0.6 was reached. Isopropyl-β-D-thiogalactopyranoside was added to cultures to induce expression, and cells were grown at 30°C shaking for 16–20 h. Cells were harvested by centrifugation and were stored at −80°C.

### Protein purification

His-tagged BT_1911 and BT_1259 purification from frozen *E. coli* cell pellets was carried out by resuspending pellets in 50 mL of lysis buffer [50 mM NaPO_4_, 300 mM NaCl, 20 mM imidazole, 10 mM 2-mercapoethanol, protease inhibitor (Roche), DNAse (Sigma), and pH 8.0]. Cells were lysed by sonication. Cell debris was pelleted by centrifugation at 25,000 × *g* for 30 min at 4°C. Lysates were run over a gravity column containing 4 mL of fresh HisPur cobalt resin (Thermo Scientific) equilibrated in wash buffer (50 mM NaPO_4_, 300 mM NaCl, 20 mM imidazole, and pH 8.0). Bound BT_1911 was washed on the column with 20 mL of wash buffer and a flow rate of ~1 mL per min and was eluted with 10 mL of elution buffer, which was wash buffer supplemented with 150 mM imidazole and 10 mM DTT and flash frozen in liquid N_2_ to prevent oxidation. Enzyme was quantified using the Qubit Protein Assay Kit (Invitrogen), and protein purity was assessed using 4%–20% SDS-PAGE gels (Thermo Scientific).

### HSDHa catalytic efficiency assays

BT_1911 kinetics were determined with slight modifications to previously established methods (48). 0.2 nM HSDH was added to pre-warmed 37°C 100 µL reactions containing 5 mM NAD^+^ in 10 mM MOPS. Bile acid substrates were included in concentrations ranging from 40 µM to 4.5 mM. Change in absorbance at 340 nm was monitored every 80 seconds in flat bottom UV-Star 96-well microplates (Greiner) from a Tecan Infinite F200 Pro Plate Reader. A standard curve of NADH was included and reaction absorbances were converted to substrate concentration since NADH is generated stoichiometrically with reaction products. Initial velocity data were plotted, and Michaelis-Menten parameters were calculated in GraphPad Prism by fitting the data to a nonlinear regression model.

### BSHa activity assay

BSH activity assays were performed as previously described (19). Briefly, the assay reacted 100 nM BSH with 9 mM bile acids for 60 min. Reactions were carried out in 0.1 mM Na phosphate, 10 mM DTT, and pH 6.0 and stopped with 50 µL of trichloroacetic acid. To determine the quantity of amino acid release, a colorimetric ninhydrin reaction was carried out. Twenty-five microliters of the quenched BSH reaction was added to 475 µL of ninhydrin buffer (0.3 mL glycerol, 0.175 mL 0.5 M Na citrate, pH 5.5, and 0.25% ninhydrin reagent) and boiled for 14 min. A standard curve of the respective conjugated amino acid or 6-APA was prepared for each assay. Absorbance was measured at 570 nm in clear flat-bottom plates in a Tecan Infinite F200 Pro Plate Reader. No detectable color change was observed, indicating that BT_1259 does not have activity on any bile acid tested.

### Animals and housing

Male and female axenic, C57BL/6J mice (aged 4–9 weeks) were purchased from the NCSU Gnotobiotic Core (Raleigh, NC) for use in gnotobiotic infection experiments. A biological safety cabinet was cold sterilized with Clidox (CAS activator: 79-14-1, base: 7758-19-2) and used to house all axenic animals and supplies used in experiments. The food, bedding, and water were all autoclaved, and other supplies that were unable to be autoclaved were cold sterilized with Clidox. Female-specific pathogen-free C57BL/6J mice (aged 4 weeks) were purchased from Jackson Laboratories for use in antibiotic-treated infection experiments. The mice were housed with autoclaved bedding and water and irradiated food. Cage changes were performed weekly in a laminar floor hood. All mice were subjected to a 12-h light and 12-h dark cycle. Animal experiments were conducted in the Laboratory Animal Facilities located on the NCSU CVM campus. The animal facilities are equipped with a full-time animal care staff coordinated by the Laboratory Animal Resources (LAR) division at NCSU. The NCSU CVM is accredited by the Association for the Assessment and Accreditation of Laboratory Animal Care International (AAALAC). Trained animal handlers in the facility fed and assessed the status of animals several times per day. Those assessed as moribund were humanely euthanized by CO_2_ asphyxiation. This protocol is approved by NC State’s Institutional Animal Care and Use Committee (IACUC).

### Gnotobiotic mouse colonization experiments

Groups of 9-week-old C57BL/6J germ-free mice (*n* = 16) were kept inside a sterilized biosafety cabinet for the duration of the experiment. All supplies were sterilized by either cold sterilization by Clidox or steam sterilization by autoclave. At day 0, all mice except the germ-free control group (*n* = 4) were challenged via oral gavage with 10^6^ CFU of *B. theta* WT (*n* = 4), 10^6^ CFU *B. theta* triple KO (*n* = 4), or both 10^6^ CFU *B. theta* WT and 10^6^ CFU *B. theta* triple KO (*n* = 4). All mouse stool tested culture negative for any bacteria before the challenge. Body weight was measured, and fecal pellets were collected throughout the experiment on days 0, 1, 3, and 7. Bacterial enumeration was performed on the fecal pellets plated on BHI-HB. Animals were humanely euthanized by CO_2_ asphyxiation followed by cervical dislocation prior to necropsy. The cecal content was collected at necropsy for bacterial enumeration. Cecal tissue snips were flash frozen in liquid nitrogen and stored at −80°C until further analysis.

### Antibiotic-treated mouse colonization experiments

Groups of 4-week-old female C57BL/6J mice (*n* = 16) were given cefoperazone (0.5 mg/mL) in drinking water *ad libitum* for 5 days. This was followed by a 2-day washout with regular drinking water (Gibco Laboratories, 15230).

At day 0, all mice except the control group (*n* = 4) were challenged via oral gavage with 10^6^ CFU of *B. theta* WT, 10^6^ *B. theta* triple KO, or both 10^6^ *B. theta* WT and 10^6^ *B. theta* triple KO. All mouse stool tested culture negative for any bacteria before the challenge. Body weight was measured every day of the experiment, and fecal pellets were collected throughout the experiment on days 0, 1, 3, 5, and 7. Bacterial enumeration was performed on the fecal pellets using Bacteroides Bile Esculin Agar (Anaerobe Systems). Animals were humanely euthanized by CO2 asphyxiation followed by cervical dislocation prior to necropsy. The ileal content and cecal content were collected at necropsy for bacterial enumeration using Bacteroides Bile Esculin Agar (Anaerobe Systems). Tissue snips were flash frozen in liquid nitrogen and stored at −80C until further analysis.

### Quantification of *B. theta* strains in co-colonization experiments

Genomic DNA was purified from *B. theta* WT and triple KO cultures using a Qiagen DNeasy UltraClean Microbial Kit. Genomic DNA from mouse fecal samples was performed using a QIAamp Fast DNA Stool Mini Kit. A standard curve using gDNA from pure culture was generated containing an increasing ratio of *B. theta* WT to triple KO DNA at a known concentration. *bshB* was used to quantify the amount of *B. theta* WT and was normalized to a gene present in both *B. theta* WT and triple KO, 16S in gnotobiotic mice, and BT_3075 in antibiotic-treated mice. The C_t_ of *bshB* was normalized to the gene present in both bacteria(2^−∆Ct^). This standard curve was then used to quantify the WT-to-triple KO ratio in mouse fecal, cecal content, and ileal content samples.

### Statistical analysis

With the exception of the RNASeq analysis, all statistical tests were performed in GraphPad Prism 8. Ordinary two-way ANOVA with Tukey’s multiple comparisons test was used to determine statistical differences between conjugated and deconjugated bile acids, while Mann-Whitney was used to determine differences between strains in the MIC data sets. Ordinary two-way ANOVA with Dunnet’s multiple comparison test was used to determine conditions statistically different from the no-bile acid control, while ordinary two-way ANOVA with Šídák’s multiple comparisons test was used to determine differences between strains in PI data sets. Ordinary one-way ANOVA with Dunnett’s multiple comparisons test was used on log-transformed data to determine conditions significantly different from the parental strain in CFU growth experiments. Unpaired *t*-test with Welch’s correction was used to determine significant differences between mid-log and stationary phase, and two-way ANOVA with Tukey’s multiple comparison test was used to determine differences in expression of genes at mid log in qPCR data. The R package DESeq2 used Wald tests with Benjamini & Hochberg correction between strains and Bonferroni correction between bile acids to identify statistically significant differentially expressed genes in the RNASeq data sets. Ordinary two-way ANOVA with Tukey’s multiple comparison test was used to determine significant differences between log transformed CFU/g of different strains in the mouse model. One-sample *t*-test was used to determine if WT strain was significantly different from a theoretical mean of 50% within the competition mouse model. Statistical significance is displayed as follows, *P < 0.05, **P < 0.01, ***P < 0.001, and ****P < 0.0001.

## RESULTS

### *B. theta* is more sensitive to deconjugated bile acids that alter membrane integrity

**Fig 1:**
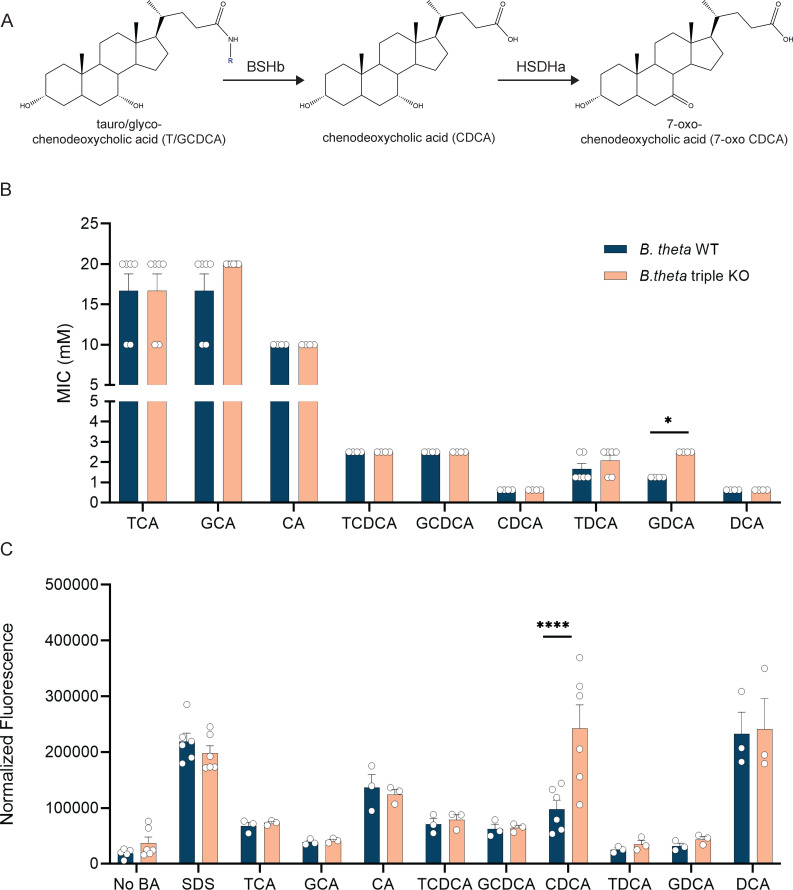
*B. theta* is more sensitive to deconjugated bile acids which are able to alter the membrane integrity. (**A**) Example of bile acid transformation carried out by BSHb and HSDHa. (**B**) WT and the triple KO strains of *B. theta* were used to determine MICs in different bile acids. Bars represent mean MICs (*n* = 2–3 biological replicates, *n* = 2 technical replicates). Error bars represent standard error. Asterisks denote significant (*P* < 0.05) by Mann-Whitney between strains. (**C**) WT and the triple KO strains were incubated with different bile acids and stained with propidium iodide. Asterisks denote significant (*P* < 0.0001) differences between strains by two-way ANOVA with Šídák’s multiple comparisons test.

To address if and how *B. theta*’s bile acid-altering enzymes are beneficial to the bacterium, seven mutant strains were generated by knocking out the genes encoding bile acid-altering enzymes *bshA* (BT_1259), *hsdhA* (BT_1911), and *bshB* (BT_2086) individually and in combination. Gram-negative bacteria are thought to be inherently more resistant to bile acids than Gram-positive bacteria as they are frequently used in media to select for Gram-negative bacteria (49). Therefore, we hypothesized that *B. theta* would be able to grow in high concentrations of bile acids. To address this, we grew *B. theta* WT in increasing concentrations of TCA, GCA, CA, TCDCA, GCDCA, CDCA, TDCA, GDCA, and DCA to determine its MIC. Since WT *B. theta* has the ability to modify bile acids (Fig. 1A), a strain of *B. theta* with all three genes encoding bile acid-altering enzymes knocked out (*∆bshA ∆hsdhA ∆bshB* referred to in this paper as the *B. theta* triple KO) was also tested against the same panel of bile acids. *B. theta* was inhibited by higher concentrations of TCA (16.6 mM), GCA (16.6 mM), and CA (10 mM), while it was more sensitive to TCDCA (2.5 mM), GCDCA (2.5 mM), CDCA (0.625 mM), TDCA (1.66 mM), GDCA (1.25 mM), and DCA (0.625 mM) (Fig. 1B). Overall, *B. theta* was only able to grow in approximately half the amount of deconjugated bile acids (CA, CDCA, and DCA) when compared with their conjugated forms (T/GCA, T/GCDCA, and T/GDCA) (Fig. 1B). The differences between conjugated and deconjugated forms were significant between T/GCA and CA in both strains (*P* < 0.0001 by two-way ANOVA with Tukey’s multiple comparison; Fig. S1A). The *B. theta* triple KO strain behaved similarly, with the only significant difference being between conjugated and deconjugated forms of T/GCA and CA (*P* < 0.0001 by two-way ANOVA with Tukey’s multiple comparison; Fig. S1B). The only difference in MICs between the WT and the triple KO strain was in the presence of GDCA (2.5 mM vs 1.25 mM, *P* < 0.05 by Mann-Whitney; Fig. 1B).

Using propidium iodide staining, we next evaluated how each bile acid affected the membrane integrity of both the WT and triple KO strain. When compared with a no-bile acid control, only deconjugated bile acids, including CA, CDCA, and DCA, were able to significantly alter the membrane in both *B. theta* WT and *B. theta* triple KO (Fig. S1C and D; *P* < 0.05, *P* < 0.01, and *P* < 0.0001 by two-way ANOVA with Dunnet’s multiple comparisons test). The only major difference between strains was in the presence of CDCA, where the triple KO was significantly more sensitive to membrane damage compared with the WT (*P* < 0.0001 by two-way ANOVA with Šídák’s multiple comparisons test; Fig. 1C). To determine if one gene is responsible for this phenotype, strains of *B. theta* with single gene deletions were also tested with CDCA in a membrane integrity assay. *B. theta* triple KO had significantly higher membrane damage than *B. theta ∆bshA* and *B. theta ∆bshB* but was not higher than *B. theta ∆hsdhA* (*P* < 0.05 and *P* < 0.001 by two-way ANOVA with Šídák’s multiple comparisons test; Fig. S1E), suggesting the missing *hsdhA* is contributing to the phenotype.

### *bshB* decreases survival in the presence of conjugated forms of CDCA and DCA

**Fig 2:**
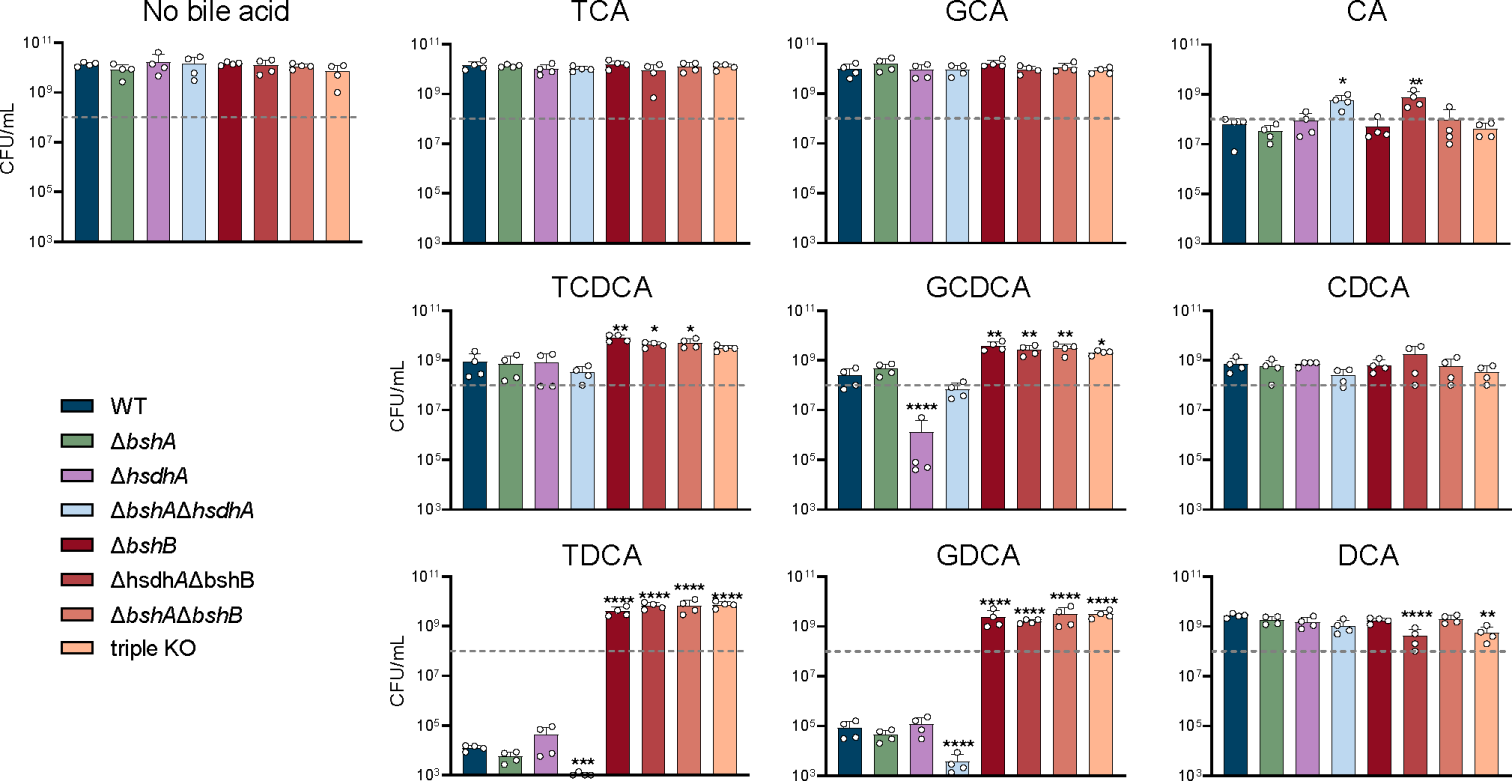
Genes encoding bile acid-altering enzymes have differential effects on *B. theta*’s fitness. Bacterial enumeration of *B. theta* WT, ∆*bshA*, ∆*hsdhA*, ∆*bshA*∆*hsdh*, ∆*bshB*, ∆*hsdhA*∆*bshB*, ∆*bshA*∆*bshB*, and triple KO strains after 24 h of growth in TYG supplemented with different bile acids. Error bars represent standard deviation from *n* ≥ 2 biological replicates. Dashed gray line represents the approximate starting CFU/mL added at 0 h. Asterisks denote significant (*P < 0.05, **P < 0.005, ***P < 0.0005, and ****P < 0.00005) differences from WT strain by one-way ANOVA.

Given the differences in both the MIC and membrane integrity assays between the *B. theta* WT and triple KO strains, we wanted to better understand how each gene encoding the bile acid-altering enzymes affects growth alone and in combination with the others. A subinhibitory concentration of each bile acid was selected based on their individual MIC. We evaluated bacterial growth via enumeration at 12 and 24 h to capture differences in growth kinetics (Fig. 2; Fig. S3). Measures at both 12 and 24 h were warranted as some bile acids increased lag phase growth of the WT strain, which showed limited growth after 12 h (Fig. S2), while other bile acids caused death between 12 and 24 h (Fig. 2; Fig. S3). Enumeration was chosen as this observed death did not correlate to a drop in OD_600_ (Fig. 2; Fig. S2).

In the no-bile acid control, all strains grew to similar levels after 24 h of growth (Fig. 2). In accordance with the lack of BSHa activity seen previously (24), Δ*bshA* had a similar growth profile to the WT strain in all bile acid conditions (Fig. 2). We also heterologously expressed and purified BSHa from *E. coli* (Fig. S4, BT_1911 is HSDHa and BT_1259 is BSHa), and it did not show any BSH or penicillin V acylase activity *in vitro*. However, strains lacking a *bshB* were significantly more resistant to TCDCA, GCDCA, TDCA, and GDCA toxicity relative to the WT strain (*P* < 0.05, *P* < 0.01, *P* < 0.001, and *P* < 0.0001 by one-way ANOVA with Dunnet’s multiple comparison test; Fig. 2). Strains containing *bshB* had growth that trended lower than strain’s missing *bshB* (Fig. 2), further supporting the role of BSHb as the only active BSH encoded by *B. theta*. These findings suggest that *bshB* may be detrimental to *B. theta’s* growth in the presence of TCDCA, GCDCA, TDCA, and GDCA.

### *B. theta* hydroxysteroid dehydrogenase acts on CDCA core bile acids

While HSDHs play an important role in the metabolism of secondary bile acids (50), they have not been directly implicated in bacterial fitness. However, Δ*hsdhA* survival was significantly reduced when grown in the presence of GCDCA when compared with the WT strain (*P* < 0.0001 by one-way ANOVA with Dunnet’s multiple comparison test; Fig. 2). This is in contrast to Δ*bshA* and Δ*bshB*, which grew similarly to WT when grown in the presence GCDCA (Fig. 2), suggesting that HSDHa may be acting on GCDCA in addition to its deconjugated form, CDCA. Since there are few examples of conjugated bile acid recognition by HSDHs in the literature (36, 51, 52), we characterized the activity and substrate range of HSDHa by heterologously expressing and purifying the enzyme to homogeneity from *E. coli* (Fig. S4). HSDHa displayed high activity on the bile acids CA, CDCA, GCDCA, and TCDCA measured via the reduction of cofactor NAD^+^ to NADH using a UV-visible spectrophotometer; however, it displayed a clear preference for CDCA (Table 1). Furthermore, the catalytic efficiency of GCDCA dehydrogenation remained high despite the glycine conjugation (Table 1). These *in vitro* observations support a mechanism of HSDHa dehydrogenation of GCDCA during *B. theta* growth. While it is not clear if this transformation reduces bile acid toxicity or adapts metabolism for persistence, taken together, these data suggest that *hsdhA* promotes survival in the presence of GCDCA.

**TABLE 1:** Catalytic efficiency measurements of the 7-α HSDH encoded by *B. theta*

	CA	CDCA	TCDCA	GCDCA
*K*_m_ (μM)	649.2 ± 167.6	203.4 ± 24.3	301.2 ± 60.4	342.2 ± 104.1
*k*_cat_ (s^−1^)	4,072.0 ± 195.3	4,580.8 ± 375.3	4,517.0 ± 681.1	4,088.8 ± 291.5

### Bile acid-altering enzymes are highly expressed in stationary phase of growth when nutrients are limited

**Fig 3:**
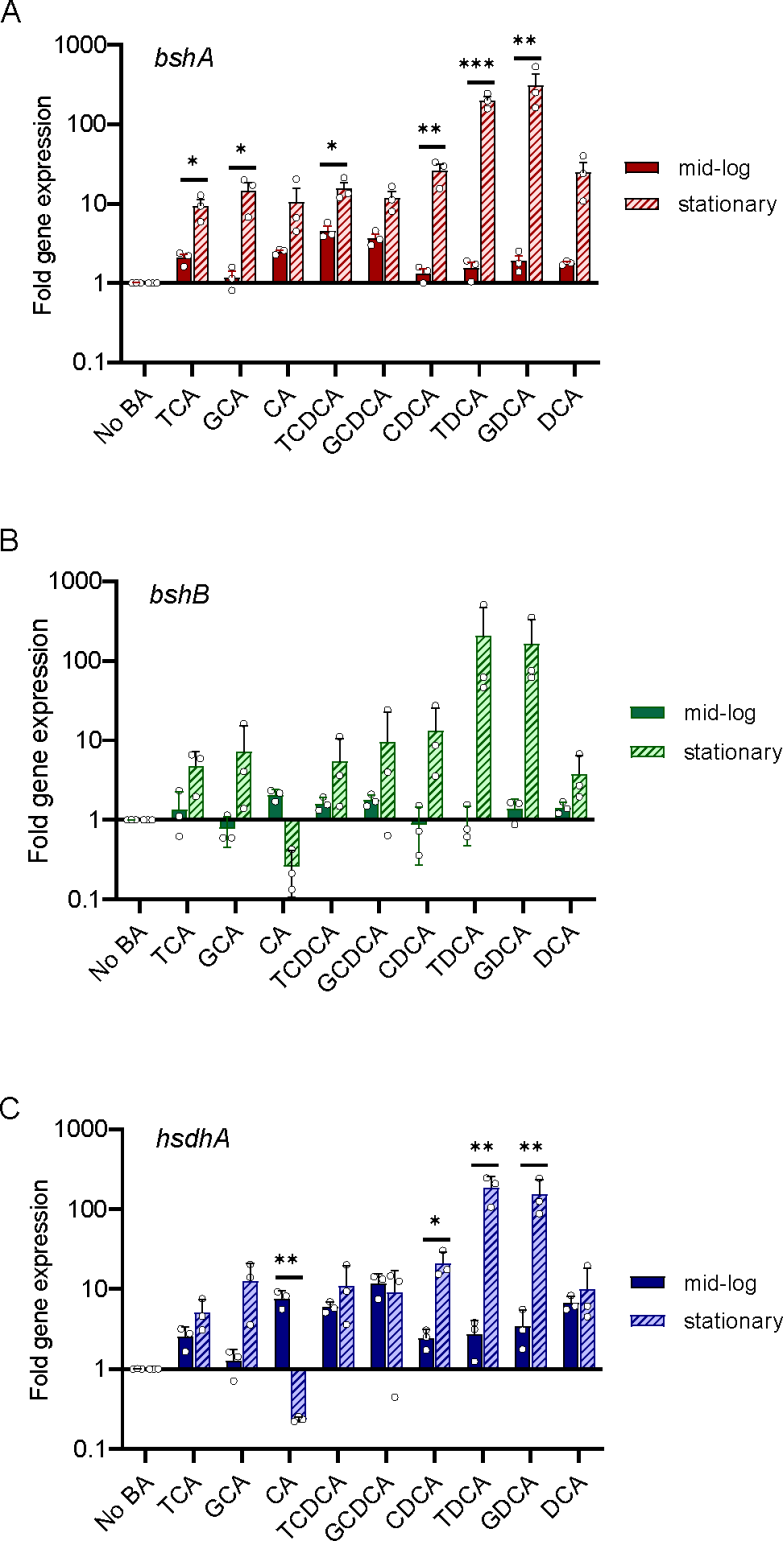
Genes encoding bile acid-altering enzymes are highly expressed in stationary phase of growth when nutrients are limited. Bars represent fold change in gene expression for (**A**) *bshA*, (**B**) *bshB*, and (**C**) *hsdhA* from mid-log and stationary phase cultures grown in different bile acids compared with growth in the absence of bile acid. Data were normalized using the ∆∆Ct method using 16S rRNA as a housekeeping gene. Error bars represent standard error between replicates (*n* = 3 biological and 3 technical replicates each). Asterisks denote significant (*P < 0.05, **P < 0.01, and ***P < 0.001) differences between mid-log and stationary phase growth stages by *t*-test.

To gain further insight into how bile acid-altering enzymes impact *B. theta*’s growth in subinhibitory concentrations of bile acids, their expression was monitored by qRT-PCR. RNA was extracted at mid-log and stationary phase from rich medium (TYG) cultures supplemented with each bile acid. Expression of *bshA* increased in stationary phase of growth compared with mid-log phase of growth in all bile acids except for CA and DCA (*P* < 0.05, *P* < 0.01, and *P* < 0.001 by unpaired *t*-test with Welch’s correction; Fig. 3A). There were no significant differences in the expression of *bshB* between mid-log and stationary phase growth (Fig. 3B). The variability between the replicates could be due to an active BSHb, but a similar trend can still be observed, where expression increases in stationary phase (Fig. 3B). Expression of *hsdhA* increased significantly in stationary phase in the presence of CDCA, TDCA, and GDCA (*P* < 0.05 and *P* < 0.01 by unpaired *t*-test with Welch’s correction) while decreasing in the presence of CA (*P* < 0.01 by unpaired *t*-test with Welch’s correction; Fig. 3C). Additionally, expression of *hsdhA* was higher than *bshA* and *bshB* in both CA and GCDCA during mid-log growth (*P* < 0.00005 by two-way ANOVA with Tukey’s multiple comparison test; Fig. S5). This high level of expression during stationary phase may be reflective of the nutrient-deprived or stressed environment.

### Metabolism of *B. theta* is impacted by bile acid-altering enzymes

**Fig 4:**
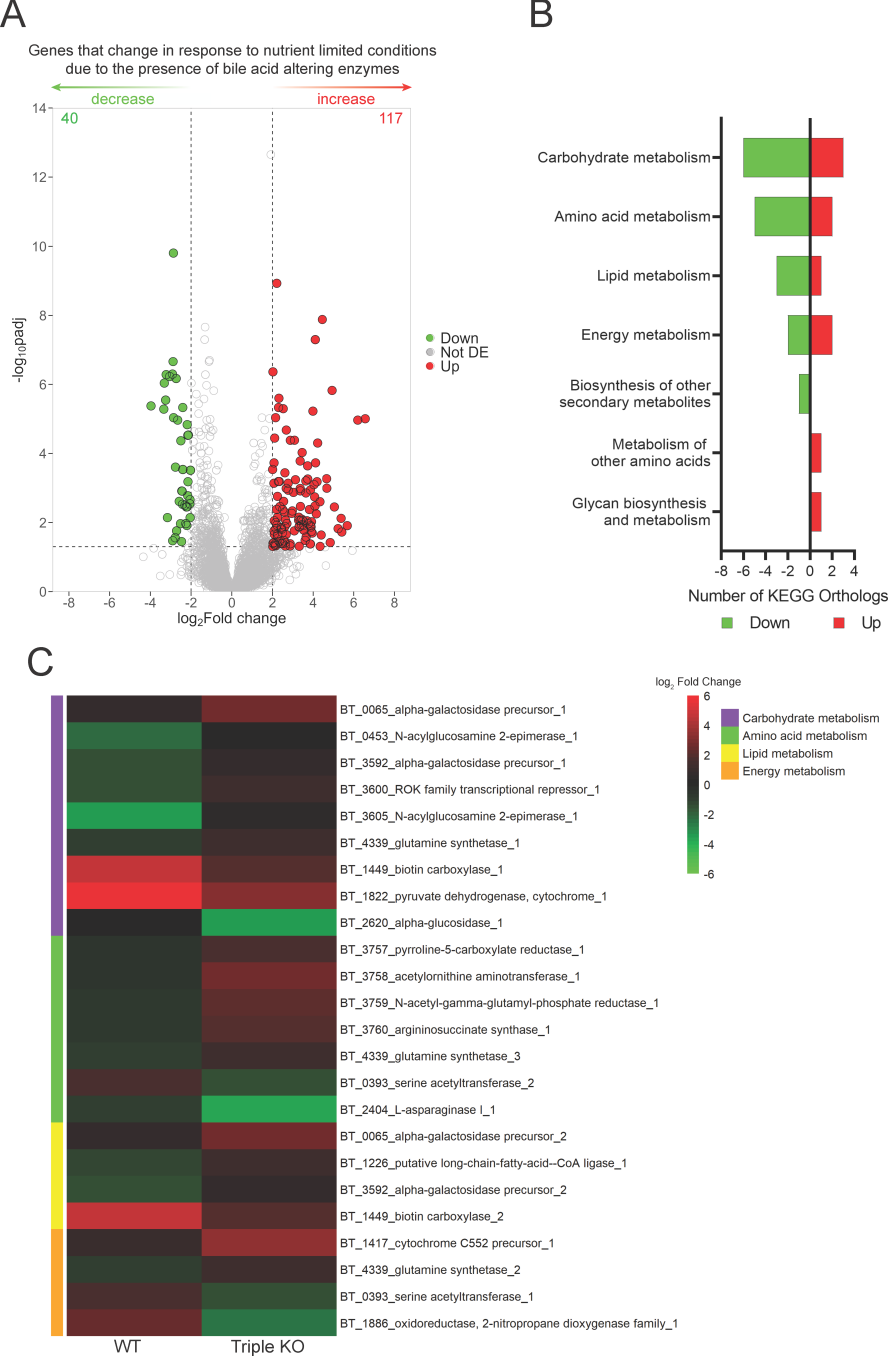
Global metabolism of *B. theta* is impacted by the absence of bile acid-altering enzymes in nutrient-limited conditions. (**A**) Volcano plot of differentially expressed genes identified by RNASeq during nutrient-limiting conditions. Points are colored when *P* < 0.05 by Wald test with Benjamini and Hochberg correction and log_2_fold change is >2 (red) or <2 (green). The number of significant genes is listed in the top corners for both directions. (**B**) KEGG orthologs detected for genes listed in (**A**). (**C**) Heatmap of differentially expressed genes present in the top four KEGG orthology categories in (**B**).

Given that genes encoding bile acid-altering enzymes are highly expressed in stationary phase of growth when nutrients are limited, we sought to determine the effect that the bile acid-altering enzymes have on the global metabolic transcriptome in nutrient-limiting conditions. We sought specifically to limit amino acids in order to determine if *B. theta* may be utilizing the amino acid liberated from bile acids by BSHb. In order to determine if the presence of bile acid-altering enzymes impacts *B. theta*’s response to these conditions, RNA was extracted and purified from WT and triple KO cultures grown to mid-log phase growth in rich (TYG) and MM, which has reduced glucose and no tryptone or yeast extract, and differential expression analysis was performed. Differential expression analysis was performed to determine how each strain responds to nutrient-limiting conditions; then, we compare these responses between the strains. The total number of genes that are increased in nutrient-limited conditions due to the presence of bile acid-altering enzymes was 117 (Up), while the number of genes that decrease in response to nutrient-limited conditions due to the presence of bile acid-altering enzymes was 40 (Down) (Fig. 4A). These are genes that changed in expression in nutrient limitation due to the genotype. To gain insight into the types of genes differentially expressed, we determined KEGG orthologs. We were able to successfully identify KEGG orthologs for 10 genes positively associated with bile acid-altering enzymes in nutrient-limited conditions and 17 genes negatively associated with bile acid-altering enzymes in nutrient-limited conditions (Fig. 4B). Carbohydrate metabolism and amino acid metabolism were the most affected metabolic pathways (Fig. 4B). Lipid and energy metabolisms were also impacted to a lesser degree. The genes identified in carbohydrate metabolism are involved in a variety of pathways. The response to nutrient limitation due to the presence of bile acid-altering enzymes shows a reduction in expression of genes involved in the metabolism of specific sugars such as galactose, glucosamine, and the amino acid glutamine whose metabolism feeds into the glyoxylate pathway, whereas there is an increase in broader enzymes including biotin carboxylase, pyruvate dehydrogenase, and alpha-glucosidase (Fig. 4C). This may indicate a change in carbohydrate preference in nutrient-limiting conditions. Amino acid metabolism was impacted primarily through an operon BT_3757-BT_3760, which is involved in arginine/ornithine metabolism, and was reduced in expression in nutrient-limited conditions due to the presence of bile acid-altering enzymes (Fig. 4C). We did not observe any changes in the gene-associated metabolism of bile acid-associated amino acids, taurine, or glycine. This was expected as there are no bile acids present from which these amino acids can be liberated. A serine acetyltransferase and L-asparaginase increased in response to nutrient limitation because the presence of bile acid-altering enzymes was also observed (Fig. 4C). Many genes within lipid metabolism are also categorized as carbohydrate metabolism with the only unique gene being a long-chain fatty acid CoA ligase which decreased in expression due to the presence of bile acid-altering enzymes (Fig. 4C). Changes in these genes may be in response to the lipids necessary for membrane homeostasis due to bile salt interaction. Additionally, these genes may interact with bile acids as bile acids help dissolve lipids during digestion. Energy metabolism, in which HSDHs may play a role in the electron transport chain, show a decrease in cytochrome C552 precursor and an increase in oxidoreductase, 2-nitropropane dioxygenase family due to the presence of bile acid-altering enzymes (Fig. 4C).

### Carbohydrate metabolism is most affected by bile acid exposure in nutrient-limited conditions

**Fig 5:**
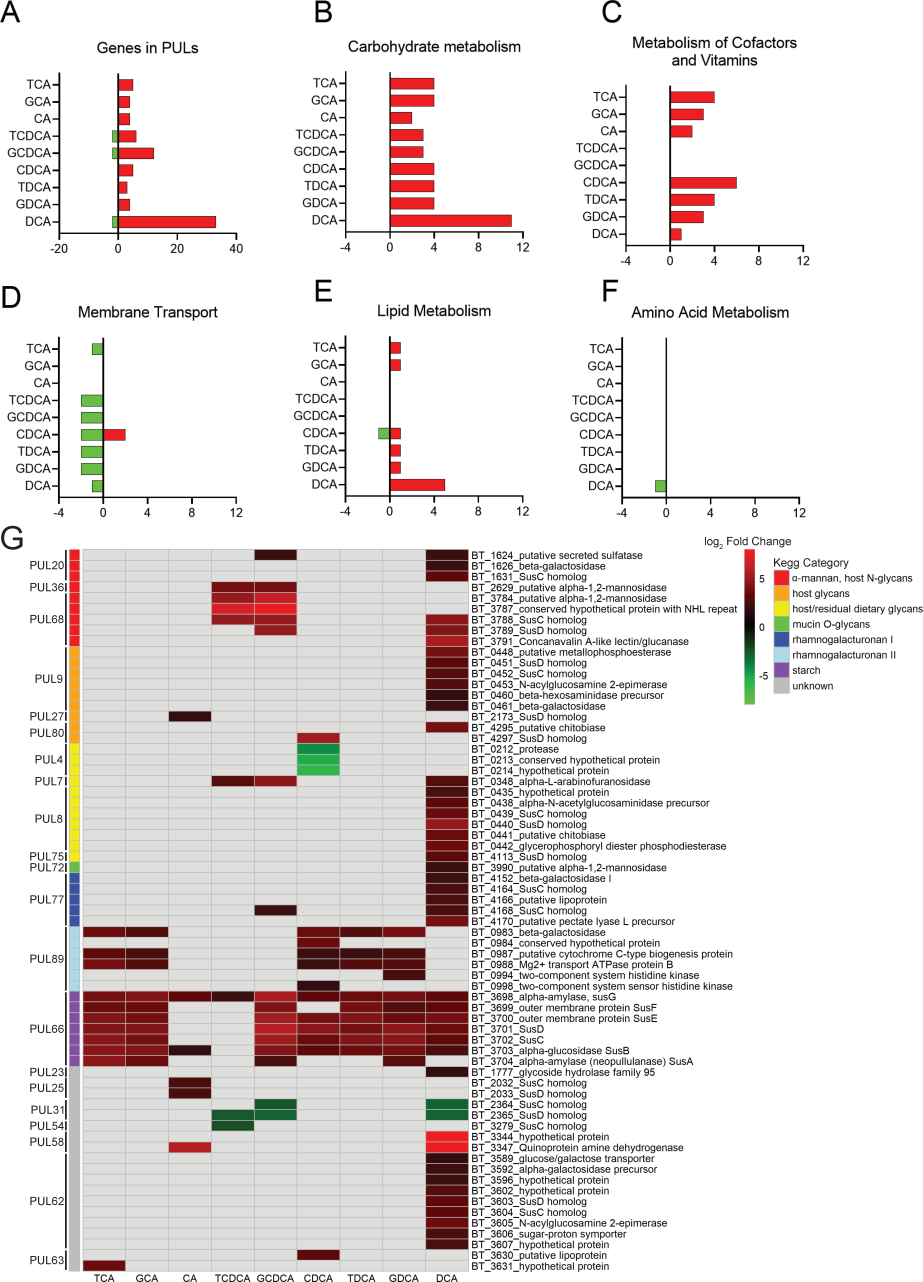
Different bile acids are able to alter *B. theta*’s metabolism in nutrient-limiting conditions. Differently expressed genes that fall under KEGG orthologs: (**A**) PUL and (**B**) carbohydrate metabolism, (**C**) metabolism of cofactors and vitamins, (**D**) membrane transport, (**E**) lipid metabolism, and (**F**) amino acid metabolism. Genes are displayed that had expression of *P* < 0.05 by Wald test with Bonferroni correction, and log_2_fold change is greater than 2 or less than −2. (**G**) Heatmap of significantly differentially expressed genes annotated as being part of a PUL where *P* < 0.05 by Wald test with Bonferroni correction and log_2_fold change > 2 or <−2. Values represent the log_2_fold change of genes that were significantly differentially expressed in nutrient-limited conditions due to the presence of a bile acid. Target substrates and gene annotation of PULs were annotated using CAZy’s PULDB (http://www.cazy.org/PULDB/) and from multiple publications for deeper annotation (40 – 47).

Since carbohydrate and amino acid metabolisms were most affected in Fig. 4 and amino acids are liberated by BSHs, we sought to determine how bile acids affect the global metabolic transcriptome of WT *B. theta* in the same nutrient-limited conditions as described previously. RNA was purified from WT cultures grown in rich or minimal media in the presence and absence of the following bile acids; TCA, GCA, CA, TCDCA, GCDCA, CDCA, TDCA, GDCA, and DCA. Differential expression analysis was done to determine which genes respond to nutrient-limiting conditions in the presence of bile acids, compared with a no-bile acid control. The number of genes differentially expressed varied by bile acid, with DCA having the most differentially expressed genes, with 59 increased in expression and 13 decreased in expression (Fig. S6). A large response of polysaccharide utilization locus-associated genes and carbohydrate metabolism was observed in the presence of most bile acids (Fig. 5A and B; Fig. S6). The majority of genes were increased in expression in nutrient-limited conditions due to the presence of a bile acid. A similar trend was observed in genes associated with the metabolism of cofactors and vitamins (Fig. 5C; Fig. S7), with the exception of TCDCA and GCDCA. Interestingly, the addition of a bile acid decreased membrane transport in nutrient-limiting conditions (Fig. 5D; Fig. S7). These genes are currently annotated vaguely as ABC transporters; some of which are designated as glycine betaine transporters (Fig. S7), potentially implicating some less defined genes as bile acid transport systems. Signal transduction increased in nutrient-limiting conditions due to the presence of bile acids (Fig. 5E; Fig. S7). Amino acid metabolism was minimally affected by the presence of bile acids, and no correlation between amino acid metabolism and bile acids, for which *B. theta* BSHb has activity on, was observed (Fig. 5F; Fig. S7).

*B. theta*’s response to nutrient limitation was impacted differently depending on the sterol core and amino acid conjugate, in both PULs (Fig. 5G) and other types of metabolism (Fig. S7). Of the 21 PULs that had genes differentially expressed, DCA showed the broadest ability to impact them by affecting 14 PULs (Fig. 5G). Bile acids primarily increased expression of genes in PULs in nutrient-limited conditions with the exceptions being PUL4, PUL31, and PUL54 that decreased in expression due to the presence of some bile acids (Fig. 5G). PUL66, responsible for *B. theta*’s ability to degrade starch, was most broadly impacted by all bile acids, which was independent of sterol core or conjugated amino acid (Fig. 5G). All genes within each PUL were not differentially expressed consistently, with some genes within the PUL responding more than others (Fig. 5G). Neither taurine nor glycine metabolism genes were impacted by the addition of bile acids (Fig. S8), but this could be due to the limited annotation in KEGG as well. To overcome this, we utilized BlastKOALA to categorize previously uncategorized genes in KEGG but were unable to find other genes involved in these pathways.

### Bile acid-altering enzymes in *B. theta* are able to impact colonization in a mouse model

**Fig 6:**
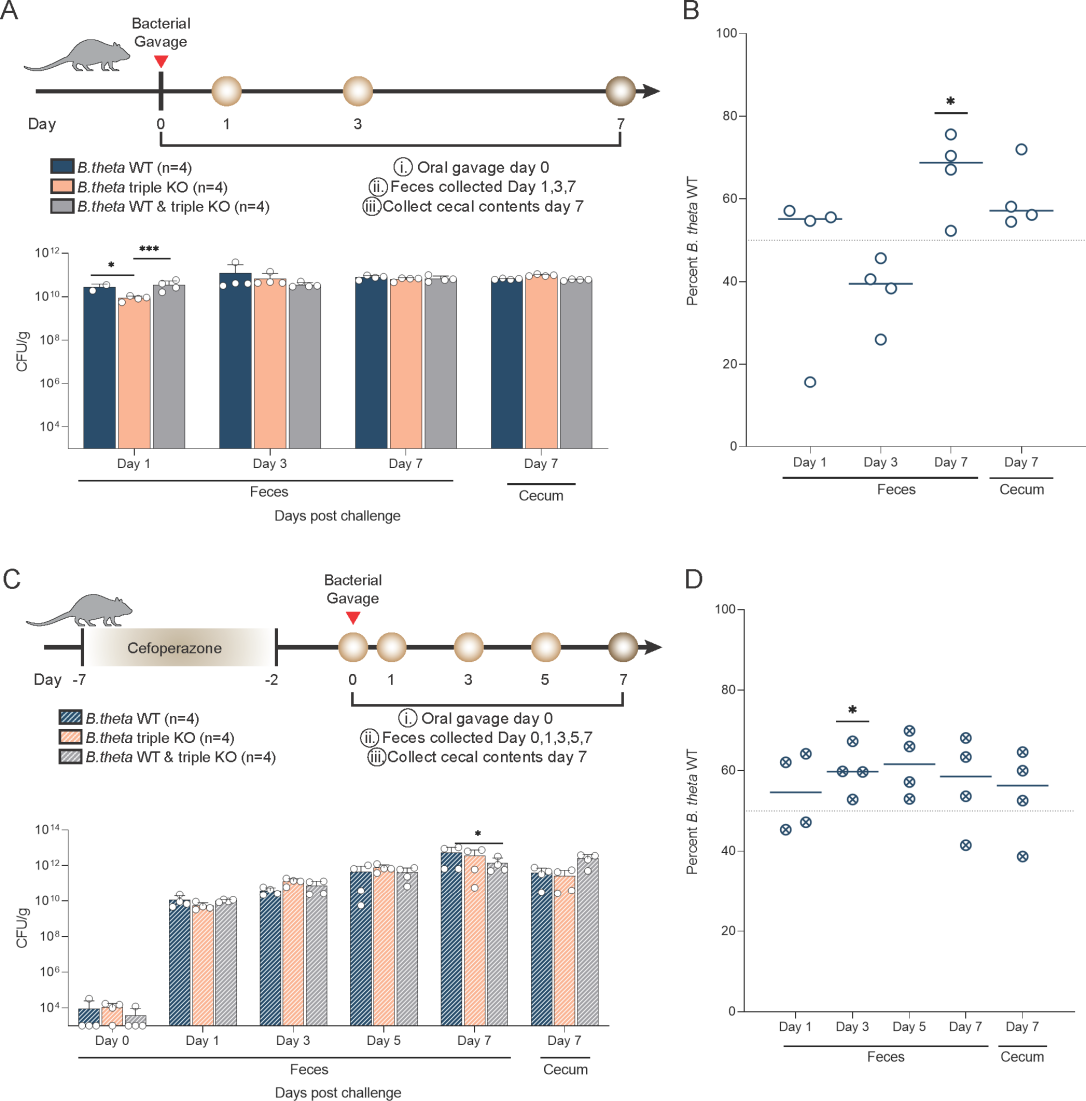
Genes encoding bile acid-altering enzymes impact colonization in a mouse model. (**A**) Experimental design for gnotobiotic mouse model. Bacterial enumeration of the fecal and cecal content in mice monocolonized with *B. theta* WT (*n* = 4), triple KO (*n* = 4), or mice co-colonized with an equal amount of *B. theta* WT and triple KO (*n* = 4). Asterisks denote significant differences (*P* < 0.05, *P* < 0.001) by two-way ANOVA with Tukey’s multiple comparison test. (**B**) qPCR of DNA extracted from fecal and cecal content of gnotobiotic mice. Asterisks denote significance differences (*P* < 0.05) via one-sample *t*-test with a theoretical mean of 50%. (**C**) Experimental design for antibiotic-treated mouse model. Bacterial enumeration of the fecal and cecal content of cefoperazone-treated mice inoculated with *B. theta* WT (*n* = 4) or triple KO (*n* = 4) or mice co-inoculated with an equal amount of *B. theta* WT and triple KO (*n* = 4). Asterisks denote significance (*P* < 0.05) by two-way ANOVA with Tukey’s multiple comparison test. (**D**) qPCR of DNA extracted from fecal or cecal content. Asterisks denote significant differences (*P* < 0.05) by one-sample *t*-test with a theoretical mean of 50%.

To further understand the benefit that *B. theta*’s bile acid-altering enzymes confer, we wanted to examine their contribution to bacterial fitness in the presence of a complex bile acid pool in a host or mouse model. Germ-free mice were monocolonized with either WT or the triple KO or co-colonized with both strains to define colonization and competition over a 7-day period (Fig. 6A). Mice monocolonized with *B. theta* WT and co-colonized with both strains had a significantly higher fecal bacterial load compared with mice monocolonized with *B. theta* triple KO at day 1 post challenge, (*P* < 0.05 and *P* < 0.001 by two-way ANOVA with Tukey’s multiple comparison; Fig. 6A). There were no differences in bacterial load in the feces at day 3 or 7 or in day 7 cecal content (Fig. 6A). However, when co-colonized, the WT strain had a significant competitive advantage compared with the triple KO at day 7 in fecal samples (*P* < 0.05 by one-sample *t*-test; Fig. 6B).

In order to measure the bacterial fitness of these strains in a more complex host, where the microbiota is intact and the bile acid pool is even more complex, we leveraged an antibiotic-treated mouse model. Cefoperazone-treated mice were challenged with either WT, the triple KO, or both strains at day 0 (Fig. 6C). Antibiotic-treated mice challenged with both strains had higher bacterial load in the cecum at day 7 compared with WT and triple KO strains alone (*P* < 0.05 by two-way ANOVA with Tukey’s multiple comparison; Fig. 6C). Similarly, the WT strain had a significant growth advantage on day 3 in fecal samples, but this advantage was lost in later timepoints potentially due to the returning microbiota (*P* < 0.05 by one-sample *t*-test Fig. 6D).

## DISCUSSION

Bile acids are host synthesized molecules that are modified by the gut microbiome. These modifications impact the host via immunity, metabolism, and the circadian rhythm (24), while also having an impact on the microbiota (19, 53). Increasing our understanding of how members of the gut microbiota are able to perform these biotransformations on bile acids and how this benefits the bacterium and surrounding community will help aid in efforts to leverage the bile acid pool to treat many diseases. *B. theta*, a predominant member of the gut microbiota, encodes three enzymes suspected to modify bile acids: BSHa, BSHb, and HSDHa (29). Previous work has focused on how these enzymes impact host physiology, but little has focused on how they benefit the bacterium itself. This study hypothesized that *B. theta* uses its BSHs and HSDH to modify bile acids to provide a fitness advantage for itself *in vitro* and *in vivo*.

Historically microbiologists have used bile acids or oxgall in media to select for Gram-negative members of the gut microbiota, so we thought that Gram-negative bacteria would be more resistant to bile acids (49). When compared with Gram-positive microbes *Lactobacillus gasseri* and *Lactobacillus acidophilus* in a previous study, *B. theta* was much more sensitive to bile acids TCDCA, TDCA, and DCA while being similarly resistant to TCA and GCA (19). In addition, lactobacilli did not show the same pattern of membrane damage, where deconjugated bile acids impacted membrane integrity more than conjugated bile acids (19). *B. theta* WT had significantly less membrane damage than *B. theta* triple KO in the presence of CDCA. This could indicate that either the HSDHa is mitigating this damage by converting CDCA to 7-oxo lithocholic acid or the BSHb is converting CDCA to a conjugated form of CDCA (14 – 16, 34).

Prior studies suggested that bacteria that encode BSHs are able to colonize the harsh gut environment better as BSHs deconjugate more toxic conjugated bile acids, making less toxic deconjugated bile acids. Our results do not support this notion and suggest that interactions between gut bacteria that encode different bile-altering enzymes and bile acids are more complex and context dependent than we first thought (19). Deconjugated bile acids, moreover than conjugated bile acids, were able to alter *B. theta*’s growth, and this was associated with impaired membrane integrity.

We went on to further characterize *B. theta*’s growth at 12 and 24 h, leveraging a panel of single, double, and triple KO mutants, and found that *bshB* was detrimental to bacterial growth in the presence of GCDCA, TDCA, and GDCA. BSHb is the primary enzyme associated with deconjugation of conjugated bile acids in *B. theta* and has activity on conjugated forms of DCA, is minimally active on conjugated forms of CDCA, and is inactive on conjugated forms of CA (24). However, this activity was observed in whole cell cultures over a 48-h period. Purified BSHb has shown limited specific activity over a short period of time (14). A recent study identifying amino acid residues required for BSH substrate selectivity found that *B. theta* BSHb is missing the selectivity loop that allows for taurine and glycine specificity (14). This may reflect a broader ability to deconjugate bile acids that are both taurine and glycine conjugated. The presence of BSHb in this study had the greatest impact on *B. theta*’s growth in the presence of bile acids that it has the highest activity on. The absence of this enzyme did provide a fitness advantage in the presence of GCDCA, TDCA, and GDCA. This could reflect the fact that DCA is more toxic than TDCA and GDCA in Fig. 1B, as well as BSHb acts on both TDCA and GDCA (24). TDCA and GDCA may be being converted into the more toxic form of DCA by this enzyme *in vitro*. Deconjugation of bile acids is detrimental to *B. theta*, and this may explain why BSHb is secreted away from the cell in extracellular vesicles (33). The extracellular secretion of BSHb may also explain why *B. theta* does not utilize taurine or glycine liberated from the bile acids it deconjugates (Fig. S8). We also observed that CDCA and DCA decreased growth in many strains lacking *bshB* (Fig. 2). *B. theta* was able to reconjugate DCA with a variety of amino acids in whole cell cultures (34). This suggests that BSHb could be reconjugating DCA with different amino acids, into a less toxic conjugated form that protects *B. theta* from further damage.

The loss of *bshA* showed a distinctly different response. Previous work has been unable to define the activity of BSHa in 50 µM of bile acid (24); however, knockouts of BSHb show some ability to deconjugate TCA and GCA at higher bile acid concentrations of 5 mM (33). In addition, this enzyme does not have a selectivity loop that is specific for taurine- and glycine-conjugated bile acids. We purified BSHa and tested its ability to deconjugate TCA, GCA, TCDCA, GCDCA, TDCA, and GDCA but were unable to measure activity (data not shown). There were no notable changes to *B. theta*’s tolerance to bile acids and growth in bile acids due to the absence of this enzyme.

Over the course of the growth experiments, *hsdhA* was able to provide a protective effect in the presence of GCDCA. Since this enzyme has not been fully characterized, we wanted to further investigate its substrate specificity and activity in the presence of TCA, GCA, CA, TCDCA, GCDCA, and CDCA. The 7α-hydroxysteroid dehydrogenase encoded by *B. theta* has been previously described to have activity on taurine- and glycine-conjugated, as well as deconjugated, forms of CA and CDCA using concentrated lysates (36). *B. theta*’s HSDHa had appreciable activity on CA, as well as taurine- or glycine-conjugated and deconjugated forms of CDCA (Table 1). The ability to act on conjugated forms of bile acids contradicts the notion that bile acids are first deconjugated before modifications to the hydroxyl group occur (54, 55). We also saw that of the three genes encoding bile acid-altering enzymes, *hsdhA* is more highly expressed in the presence of CA, TCDCA, GCDCA, and DCA (Fig. S4). HSDHa may be providing a protective effect by changing the structure of the conjugated bile acid before BSHb is able to deconjugate it, where that deconjugation is detrimental to *B. theta’s* growth. Additionally, the protective effect of HSDHa may be derived from the detoxification of bile acids through their conversion to 7-oxo forms (22).

The absence of bile acid-altering enzymes led to global metabolic changes in nutrient-limited conditions independent of bile acid supplementation. We saw large shifts in response to nutrient-limited conditions including genes important for carbohydrate metabolism, amino acid metabolism, lipid metabolism, and energy metabolism between the WT and triple KO strains. Since nutrient-limiting conditions lacked amino acids, we expected changes in amino acid metabolism. We did observe a decrease in arginine/ornithine metabolism in nutrient-limited conditions due to the presence of bile acid-altering enzymes. Ornithine metabolism is important for gut pathogen colonization including *C. difficile* physiology and pathogenesis (56, 57). However, a limitation of our study is that we did not test for changes in the global metabolic transcriptome in the knockout strain due to bile acids and therefore cannot determine if the absence of bile acid-altering enzymes impacts amino acid metabolism of amino acids freed by bile salt hydrolases. This suggests that encoding bile acid-altering enzymes could have far reaching metabolic impacts that affect not only the bacterium that encodes them but gut pathogens as well. The addition of bile acids greatly impacted *B. theta*’s global metabolic response to nutrient limitation, and this was dependent on the sterol core and conjugated amino acid. Many carbohydrate utilization genes were increased in expression in nutrient-limited conditions due to the presence of a bile acid. Bile acid stress has been shown *in vivo* to alter the microbial metabolism, including carbohydrate metabolism, in the cecum (58). However, the impact bile acids have on each member of the microbiota has not been well studied.

We also investigated the potential for the amino acid being freed by *B. theta* BSHs, taurine or glycine, being used as a nutrient, or for the electron lost during dehydroxylation to feed into energy metabolism. However, we primarily observed an increase in genes important for carbohydrate metabolism and associated with PULs, not amino acid or energy metabolism. This could be due to the limited annotated genes in that pathway. The primary carbohydrate source used in both the minimal and rich media is glucose. Bile acids directly interact with carbohydrates as they can bind to fiber, with certain fiber sources such as lignin binding more so than bran (59). Fiber intake also has impacts on the composition of the bile acid pool (60). Considering the strong response in carbohydrate metabolism in the presence of bile acids, *B. theta* may be using different carbohydrates to exploit bile acid-fiber binding and thus altering the types of fiber available in the gut. If bile acids are bound to fiber, they can’t be reabsorbed through enterohepatic circulation and the host’s homeostasis will synthesize more bile acids from cholesterol (61). This has been proposed as a way to reduce cholesterol, and our findings may indicate that the host microbiome, more specifically *B. theta*, might influence this interaction. PULs were also impacted in nutrient-limited conditions due to the presence of a bile acid. These PULs targeted a variety of substrates ranging from starches to host glycans. DCA, an inflammatory bile acid (62), impacted 14 of 21 differentially expressed PULs including ones that target host glycans and mucins. This suggests *B. theta* may be taking advantage of the inflammatory nature of this bile acid.

The native environment of *B. theta* is the mammalian gut, an environment that has a diverse pool of bile acids, and as such, it is not only coming into contact with one single bile acid at a time. To address this limitation and determine if the presence of these genes is required for colonization or creates a competitive advantage in a complex gut environment, we first used a gnotobiotic mouse model. In mono-colonization experiments, there were no significant differences 3 days post challenge. This is consistent with a work which showed no significant difference between CFUs in WT vs triple KO strains of *B. theta* in the feces after 2 weeks (8). While slight differences in bacterial load were observed 1 day after challenge in the monocolonized mice, this difference was not recapitulated in the competition model. To determine if these enzymes impact colonization alongside a more complex microbiota, this experiment was repeated using mice treated with cefoperazone to open up a niche for *B. theta* to colonize. Again, while we observed slight advantages in the WT over the triple KO strain, bile acid-altering enzymes did not allow one strain to overwhelm the other. Since the competitive advantage between the WT and triple KO was very slight in both mouse models and the complex bile acid pools do not mirror the ones seen in the *in vitro* assays, we did not complement strains to determine each gene’s individual role is this phenotype. Although we did not complement strains, we used RNASeq data to evaluate polar mutations and found a total 10 genes differentially expressed (~0.2% of genes) when comparing WT and triple KO gene expression in both rich and minimal media. Of these genes, the closest to a knockout was >45 kbp away, suggesting very minimal to no polar mutations.

Changes to the bile acid pool are associated with a wide variety of diseases including *C. difficile* infection (CDI) (10), inflammatory bowel disease (11), metabolic syndrome (12), diabetes (26), hepatic fibrosis (25), different cancers (12, 13), and more recently depression (63). Research on the enzymes that modify the bile acid pool is expanding with the recent finding that BSHs are associated with reconjugation of bile acids or MCBAs (15, 16, 34). While *B. theta* was associated with the production of MCBAs (34), the specific enzyme that carries out this function has not been determined. Defining the mechanisms by which bacteria modify the bile acid pool will aid in our understanding on how to rationally manipulate the bile acid pool and the microbiota to treat many GI diseases.

## Data Availability

Raw sequences have been deposited in the Sequence Read Archive (SRA) under BioProject ID PRJNA986925. Source data are provided within each supplemental file. Other data and biological materials are available from the corresponding author upon reasonable request.

## SUPPLEMENTAL MATERIAL

Fig. S1 spectrum.03576-23-s0001.tif Genes encoding bile acid altering enzymes impact both MIC and membrane integrity of *B. theta*.

Fig. S2 spectrum.03576-23-s0002.tif Growth curves of WT *B. theta* in different bile acids with varying concentrations.

Fig. S3 spectrum.03576-23-s0003.tif Genes encoding bile acid altering enzymes have minimal effects on *B. theta*'s fitness at 12 hours.

Fig. S4 spectrum.03576-23-s0004.tif Isolation of purification of HSDH and BSHa.

Fig. S5 spectrum.03576-23-s0005.tif
*hsdhA* is highly expressed in some bile acid conditions during mid-log phase of growth.

Fig. S6 spectrum.03576-23-s0006.tif Bile acids differentially impact the *B. theta* transcriptome in response to nutrient limiting conditions.

Fig. S7 spectrum.03576-23-s0007.tif Bile acids impact multiple *B. theta* metabolic pathways in nutrient limited conditions.

Fig. S8 spectrum.03576-23-s0008.tif
*B. theta* taurine and glycine metabolism gene expression does not change in the presence of different bile acids in nutrient limiting conditions.

Supplemental legends spectrum.03576-23-s0009.docx Legends to Fig. S1 to S8 and Table S1.

Table S1 spectrum.03576-23-s0010.xlsx Oligonucleotides used in this study.

ASM does not own the copyrights to Supplemental Material that may be linked to, or accessed through, an article. The authors have granted ASM a non-exclusive, world-wide license to publish the Supplemental Material files. Please contact the corresponding author directly for reuse.
