# Supplementary figures and images for: Loss of Bacteroides thetaiotaomicron bile acid-altering enzymes impacts bacterial fitness and the global metabolic transcriptome
Source: Microbiol Spectr. 2023 Nov 29;12(1):e03576-23. doi: 10.1128/spectrum.03576-23 (PMC10783122; doi:10.1128/spectrum.03576-23)

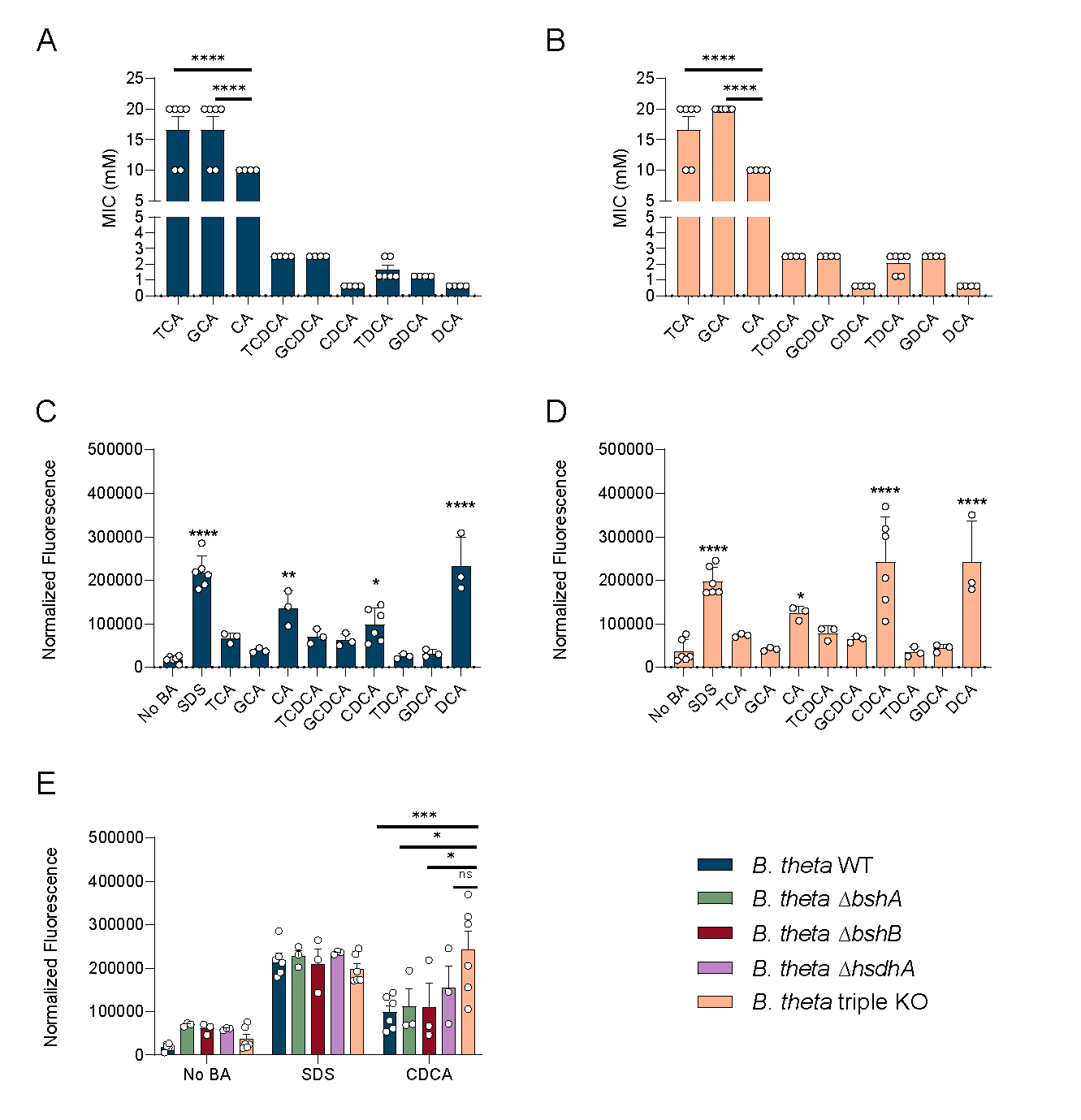

Supplement: Fig. S1 — Genes encoding bile acid altering enzymes impact both MIC and membrane integrity of B. theta. [file spectrum.03576-23-s0001.tif]

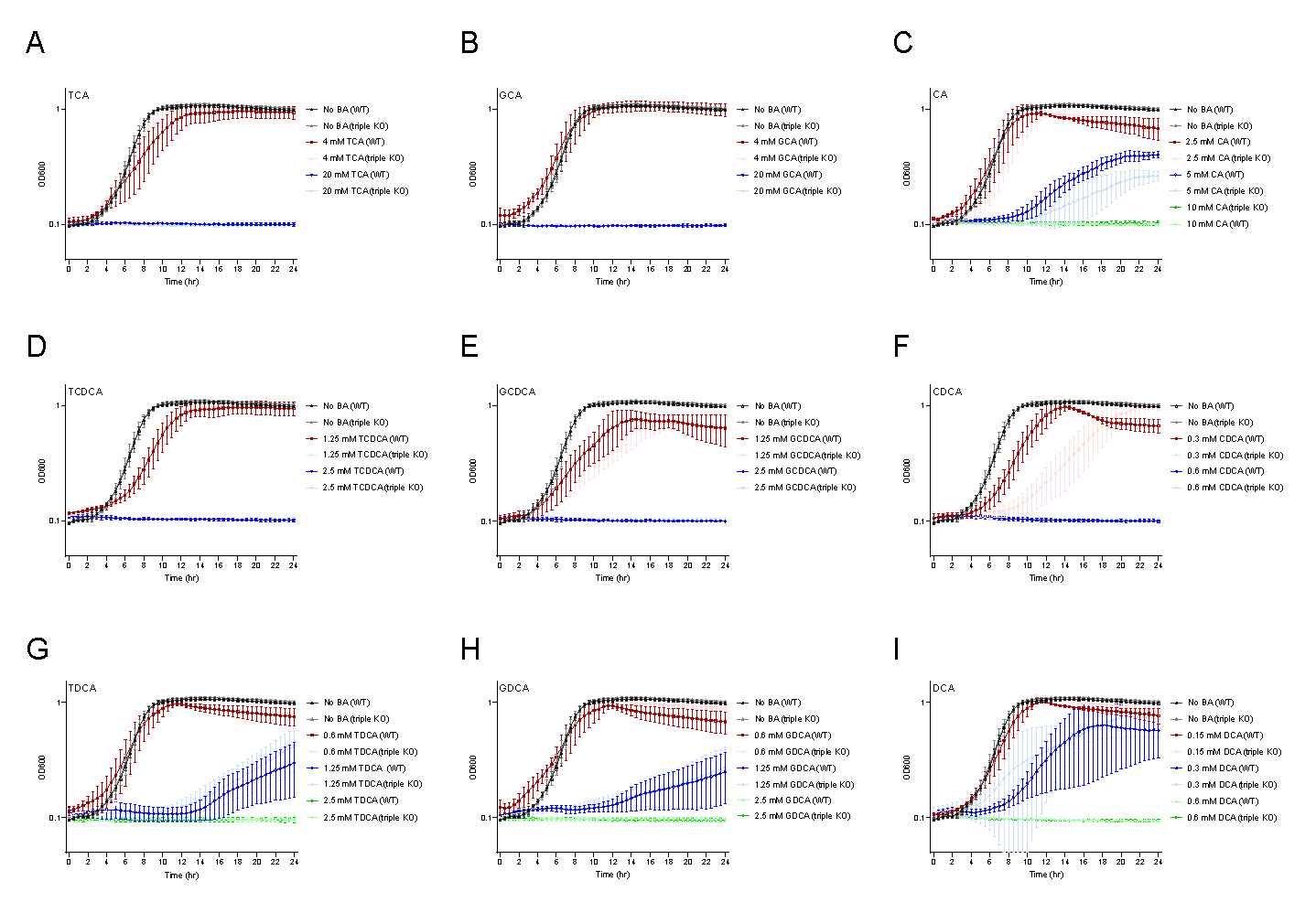

Supplement: Fig. S2 — Growth curves of WT B. theta in different bile acids with varying concentrations. [file spectrum.03576-23-s0002.tif]

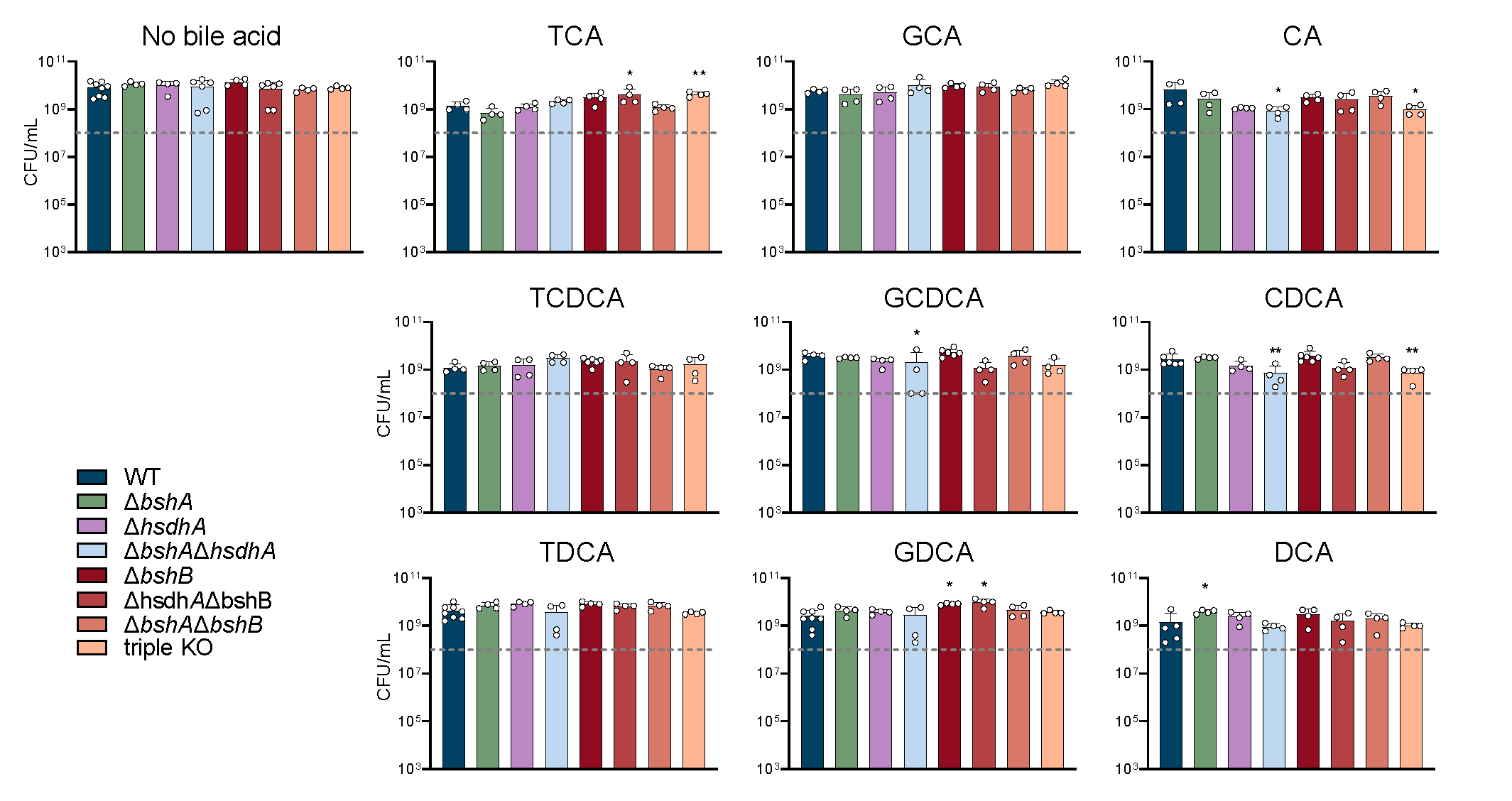

Supplement: Fig. S3 — Genes encoding bile acid altering enzymes have minimal effects on B. theta's fitness at 12 hours. [file spectrum.03576-23-s0003.tif]

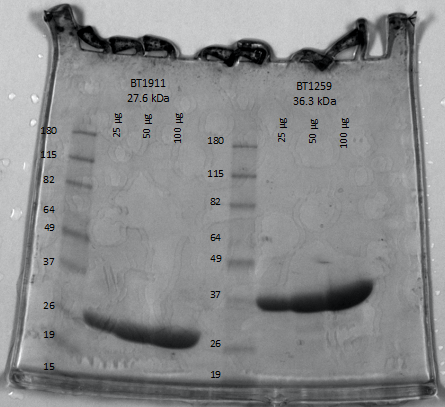

Supplement: Fig. S4 — Isolation of purification of HSDH and BSHa. [file spectrum.03576-23-s0004.tif]

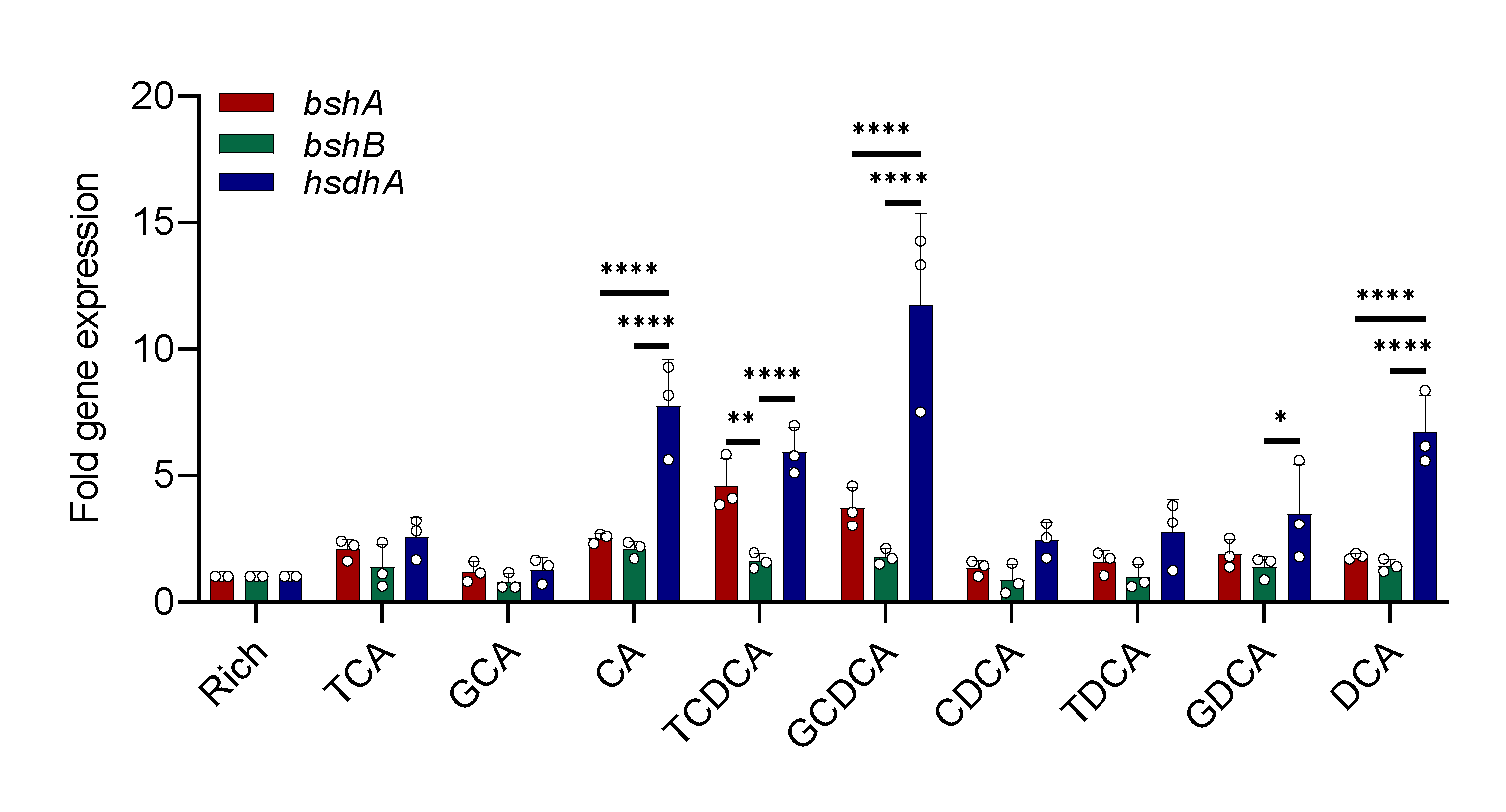

Supplement: Fig. S5 — hsdhA is highly expressed in some bile acid conditions during mid-log phase of growth. [file spectrum.03576-23-s0005.tif]

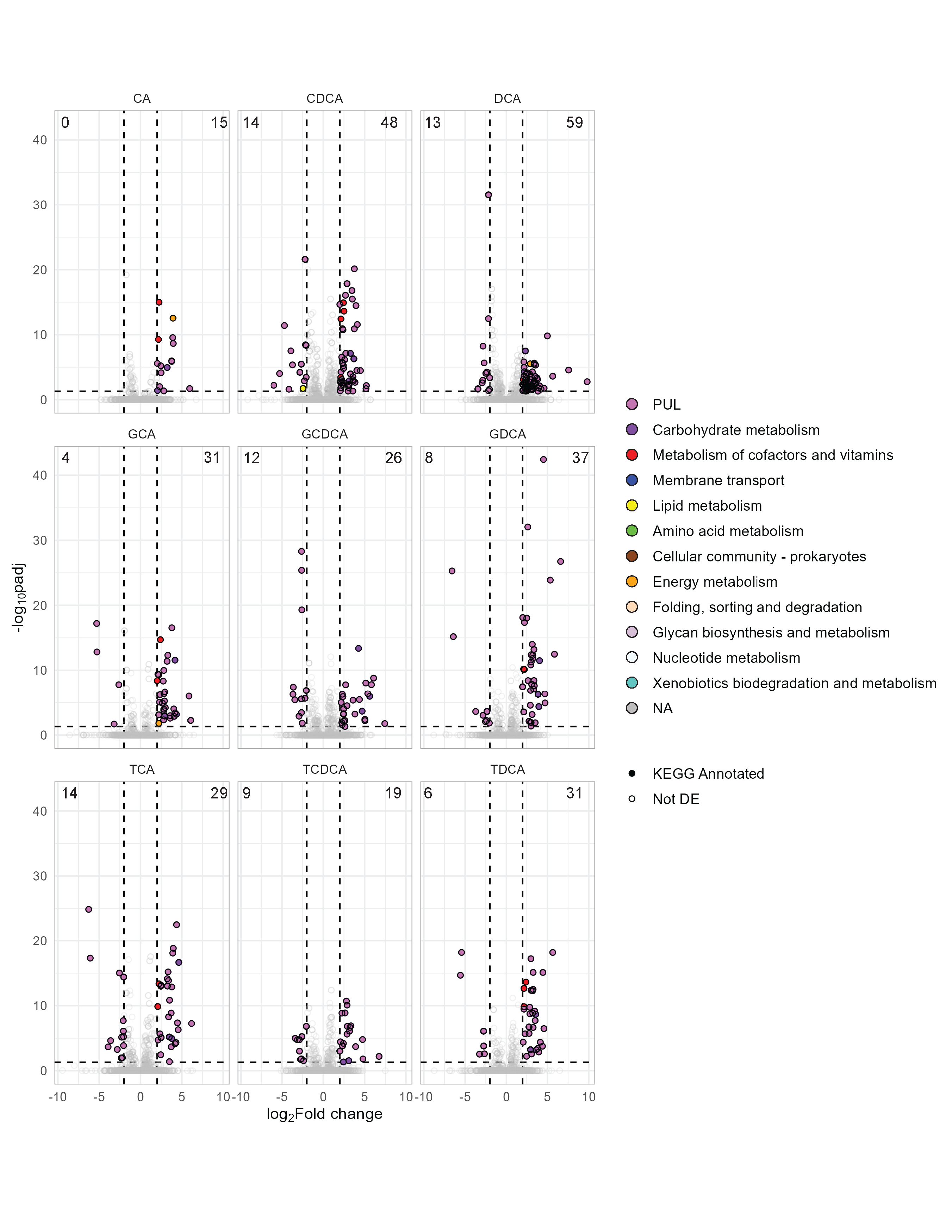

Supplement: Fig. S6 — Bile acids differentially impact the B. theta transcriptome in response to nutrient limiting conditions. [file spectrum.03576-23-s0006.tif]

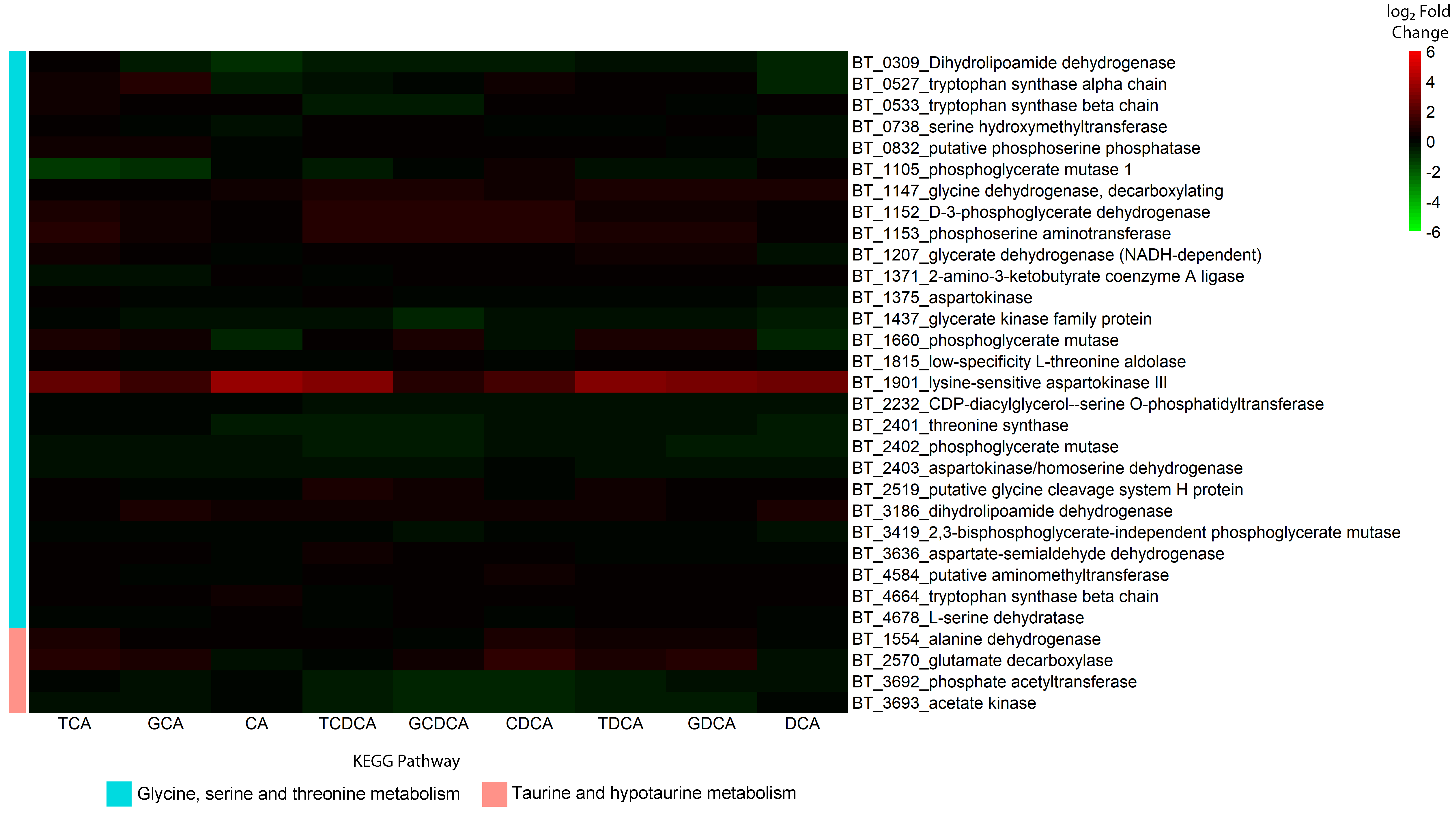

Supplement: Fig. S8 — B. theta taurine and glycine metabolism gene expression does not change in the presence of different bile acids in nutrient limiting conditions. [file spectrum.03576-23-s0008.tif]
