# Supplementary material for: Loss of Bacteroides thetaiotaomicron bile acid-altering enzymes impacts bacterial fitness and the global metabolic transcriptome
Source: Microbiol Spectr. 2023 Nov 29;12(1):e03576-23. doi: 10.1128/spectrum.03576-23 (PMC10783122; doi:10.1128/spectrum.03576-23)
Supplement: Supplemental legends — Legends to Fig. S1 to S8 and Table S1. [file spectrum.03576-23-s0009.docx]

**Supplemental Figures**

**Supplemental Figure 1. Genes encoding bile acid altering enzymes impact both MIC and membrane integrity of *B. theta*.**

*B. theta* WT (A) and triple KO (B) strains were used to determine MICs in different bile acids. Bars represent mean MICs from (n = 2-3 biological replicates, n=2 technical replicates). Error bars represent standard error. Asterisks denote significance (p<0.0001) between conjugated and deconjugated by two way ANOVA with Tukey’s multiple comparisons test. *B. theta* WT (C) and triple KO **(**D) strains were incubated with different bile acids and stained with propidium iodide. Asterisks denote significant by two-way ANOVA with Dunnet’s multiple comparison test (*p<0.05;** p<0.01; **** p<0.0001) differences between the condition and the no bile acid or no BA control. (E) WT, *∆bshA, ∆bshB, ∆hsdhA,* and the triple KO strains were incubated with CDCA and stained with propidium iodide. Asterisks denote significant (* p<0.05; *** p<0.0005) differences between each strain by two-way ANOVA with Tukey’s multiple comparison test.

**Supplemental Figure 2. Growth curves of WT *B. theta* in different bile acids with varying concentrations.**

WT and triple KO strains of *B. theta* were grown for 24 hr in TYG supplemented with different bile acids in varying concentrations. Error bars represent SD from n=3 biological replicates, n=2 technical replicates.

**Supplemental Figure 3. Genes encoding bile acid altering enzymes have minimal effects on *B. theta*’s fitness at 12 hours.**

WT and knockout strains of *B. theta* were grown for 12 hr in TYG supplemented with different bile acids. Error bars represent SD from n=2 biological replicates, n= 2 technical replicates. Dashed grey line represents the approximate starting CFU/mL added at 0 hr. Asterisks denote significant (p<0.05, p<0.01) differences from WT by one-way ANOVA with Dunnet’s multiple comparison test.

**Supplemental Figure 4. Isolation of purification of HSDH and BSHa.**

SDS PAGE gel (4-20%) of purified His-tagged BT_1911 (27.9 kDa) and BT_1259 (36.6 kDa) stained with Coomassie blue for visualization. 25 μg, 50 μg, and 100 μg of each protein was run alongside ladder labelled in kDa.

**Supplemental Figure 5. *hsdhA* is highly expressed in some bile acid conditions during mid-log phase of growth.**

Bars represent fold change in gene expression for genes encoding bile acid altering enzymes in different bile acids compared to expression in the absence of bile acid. Data was normalized using the ∆∆Ct method using 16S rRNA as a housekeeping gene. Error bars represent standard error between replicates (n=3 biological, 3 technical replicates) each. Asterisks denote significant (* p<0.05; ** p<0.01; **** p<0.0001) differences between genes in each bile acid.

by 2-way ANOVA with Tukey’s multiple comparison test.

**Supplemental Figure 6. Bile acids differentially impact the *B. theta* transcriptome in response to nutrient limiting conditions.**

Volcano plot of differentially expressed genes classified as a PUL or by KEGG orthology where p<0.05 by Wald test with Bonferroni correction and log_2_fold change > 2 or < -2 are plotted with a color. Number of genes increased in expression (top right corner) and decreased in expression (top left corner) are listed.

**Supplemental Figure 7. Bile acids impact multiple *B. theta* metabolic pathways in nutrient limited conditions.**

Heatmap of significantly differentially expressed genes with KEGG annotations where p<0.05 by Wald test with Bonferroni correction and log_2_fold change > 2 or < -2. Values represent the log_2_ fold change of genes that were significantly differentially expressed in nutrient limited conditions due to the presence of a bile acid.

**Supplemental Figure 8. *B. theta* taurine and glycine metabolism gene expression does not change in the presence of different bile acids in nutrient limiting conditions.**

Genes annotated in KEGG to be involved in taurine or glycine metabolism are listed. Values represent the log_2_ fold change in response to the addition of different bile acids in rich vs minimal cultures. No genes listed were found to be significant by Wald test with Bonferroni correction in any condition.

**Supplemental Table 1. Oligonucleotides used in this study.**
